# Supplementary material for: High-throughput discovery of genetic determinants of circadian misalignment
Source: PLoS Genet. 2020 Jan 13;16(1):e1008577. doi: 10.1371/journal.pgen.1008577 (PMC6980734; doi:10.1371/journal.pgen.1008577)
Supplement: S1 Table — (DOCX) [file pgen.1008577.s005.docx]

**S1 Table. Onset times of wild type mice from visual assessment**

| **Visual** | **Onset** | | | | | | | | | | | | | |
| --- | --- | --- | --- | --- | --- | --- | --- | --- | --- | --- | --- | --- | --- | --- |
| **Onset**  **Center** |  | **10** | **11** | **12** | **13** | **14** | **15** | **16** | **17** | **18** | **19** | **20** | **21** | **22** |
| **WTSI** | **Activity** | **0** | **12** | **194** | **270** | **13** | **1** | **0** | **0** | **0** | **0** | **0** | **0** | **0** |
|  | **Food** | **1** | **40** | **145** | **186** | **80** | **19** | **4** | **0** | **0** | **0** | **0** | **0** | **0** |
| **ICS** | **Activity** | **1** | **25** | **284** | **4** | **0** | **0** | **0** | **0** | **0** | **0** | **0** | **0** | **0** |
|  | **Food** | **0** | **48** | **227** | **105** | **33** | **5** | **3** | **0** | **0** | **0** | **0** | **0** | **0** |
| **RBRC** | **Activity** | **0** | **1** | **20** | **96** | **123** | **37** | **0** | **0** | **0** | **0** | **0** | **0** | **0** |
|  | **Food** | **0** | **10** | **44** | **92** | **68** | **13** | **0** | **0** | **0** | **0** | **0** | **0** | **0** |
| **TCP** | **Activity** | **0** | **2** | **50** | **67** | **0** | **0** | **0** | **0** | **0** | **0** | **0** | **0** | **0** |
|  | **Food** | **1** | **7** | **24** | **21** | **22** | **9** | **0** | **0** | **0** | **0** | **0** | **0** | **0** |
| **HMGU** | **Activity** | **0** | **0** | **965** | **33** | **3** | **0** | **0** | **0** | **0** | **0** | **0** | **0** | **0** |
|  | **Food** | **14** | **69** | **503** | **228** | **90** | **36** | **13** | **0** | **0** | **0** | **0** | **0** | **0** |
