# Supplementary material for: High-throughput discovery of genetic determinants of circadian misalignment
Source: PLoS Genet. 2020 Jan 13;16(1):e1008577. doi: 10.1371/journal.pgen.1008577 (PMC6980734; doi:10.1371/journal.pgen.1008577)
Supplement: S2 Table — (DOCX) [file pgen.1008577.s006.docx]

**S2 Table. Peak phases of wild type mice from visual assessment**

| **Visual** | **Phase** | | | | | | | | | | | | | |  |
| --- | --- | --- | --- | --- | --- | --- | --- | --- | --- | --- | --- | --- | --- | --- | --- |
| **Phase**  **Center** |  | **10** | **11** | **12** | **13** | **14** | **15** | **16** | **17** | **18** | **19** | **20** | **21** | **22** | **23** |
| **WTSI** | **Activity** | **0** | **0** | **19** | **96** | **196** | **89** | **52** | **23** | **12** | **1** | **1** | **1** | **0** | **0** |
|  | **Food** | **0** | **0** | **10** | **35** | **84** | **130** | **99** | **84** | **31** | **2** | **0** | **0** | **0** | **0** |
| **ICS** | **Activity** | **0** | **0** | **142** | **75** | **57** | **25** | **8** | **5** | **2** | **0** | **0** | **0** | **0** | **0** |
|  | **Food** | **0** | **1** | **49** | **57** | **84** | **97** | **70** | **42** | **13** | **5** | **0** | **1** | **1** | **0** |
| **RBRC** | **Activity** | **0** | **0** | **2** | **29** | **54** | **96** | **59** | **27** | **7** | **2** | **0** | **1** | **0** | **0** |
|  | **Food** | **0** | **0** | **3** | **33** | **71** | **78** | **26** | **9** | **6** | **1** | **0** | **0** | **0** | **0** |
| **TCP** | **Activity** | **0** | **0** | **1** | **14** | **28** | **22** | **25** | **12** | **9** | **6** | **1** | **1** | **0** | **0** |
|  | **Food** | **0** | **0** | **0** | **4** | **13** | **16** | **19** | **26** | **6** | **0** | **0** | **0** | **0** | **0** |
| **HMGU** | **Activity** | **0** | **0** | **78** | **274** | **247** | **173** | **134** | **70** | **19** | **5** | **1** | **0** | **0** | **0** |
|  | **Food** | **0** | **0** | **4** | **30** | **81** | **119** | **192** | **153** | **129** | **70** | **49** | **57** | **53** | **16** |
