# Supplementary material for: High-throughput discovery of genetic determinants of circadian misalignment
Source: PLoS Genet. 2020 Jan 13;16(1):e1008577. doi: 10.1371/journal.pgen.1008577 (PMC6980734; doi:10.1371/journal.pgen.1008577)
Supplement: S3 Table — (DOCX) [file pgen.1008577.s007.docx]

**S3 Table. Onset times of wild type mice from machine learning algorithm**

| **Auto** | **Onset** | | | | | | | | | | | | | |
| --- | --- | --- | --- | --- | --- | --- | --- | --- | --- | --- | --- | --- | --- | --- |
| **Onset**  **Center** |  | **10** | **11** | **12** | **13** | **14** | **15** | **16** | **17** | **18** | **19** | **20** | **21** | **22** |
| **WTSI** | **Activity** | **0** | **0** | **212** | **238** | **40** | **0** | **0** | **0** | **0** | **0** | **0** | **0** | **0** |
|  | **Food** | **0** | **1** | **257** | **133** | **84** | **0** | **0** | **0** | **0** | **0** | **0** | **0** | **0** |
| **ICS** | **Activity** | **4** | **128** | **163** | **18** | **1** | **0** | **0** | **0** | **0** | **0** | **0** | **0** | **0** |
|  | **Food** | **0** | **0** | **228** | **124** | **69** | **0** | **0** | **0** | **0** | **0** | **0** | **0** | **0** |
| **RBRC** | **Activity** | **0** | **0** | **0** | **97** | **180** | **0** | **0** | **0** | **0** | **0** | **0** | **0** | **0** |
|  | **Food** | **0** | **0** | **57** | **109** | **60** | **0** | **0** | **0** | **0** | **0** | **0** | **0** | **0** |
| **TCP** | **Activity** | **0** | **2** | **49** | **67** | **15** | **0** | **0** | **0** | **0** | **0** | **0** | **0** | **0** |
|  | **Food** | **0** | **0** | **26** | **30** | **38** | **0** | **0** | **0** | **0** | **0** | **0** | **0** | **0** |
| **HMGU** | **Activity** | **1** | **1** | **902** | **99** | **1** | **0** | **0** | **0** | **0** | **0** | **0** | **0** | **0** |
|  | **Food** | **1** | **52** | **354** | **239** | **168** | **114** | **24** | **1** | **0** | **0** | **0** | **0** | **0** |
