# Supplementary material for: High-throughput discovery of genetic determinants of circadian misalignment
Source: PLoS Genet. 2020 Jan 13;16(1):e1008577. doi: 10.1371/journal.pgen.1008577 (PMC6980734; doi:10.1371/journal.pgen.1008577)
Supplement: S4 Table — (DOCX) [file pgen.1008577.s008.docx]

**S4 Table. Peak phases of wild type mice from machine learning algorithm**

| **Auto** | **Phase** | | | | | | | | | | | | | |
| --- | --- | --- | --- | --- | --- | --- | --- | --- | --- | --- | --- | --- | --- | --- |
| **Phase**  **Center** |  | **10** | **11** | **12** | **13** | **14** | **15** | **16** | **17** | **18** | **19** | **20** | **21** | **22** |
| **WTSI** | **Activity** | **0** | **0** | **0** | **11** | **203** | **230** | **39** | **7** | **0** | **0** | **0** | **0** | **0** |
|  | **Food** | **0** | **0** | **36** | **101** | **170** | **136** | **29** | **0** | **0** | **0** | **0** | **0** | **0** |
| **ICS** | **Activity** | **0** | **0** | **9** | **121** | **99** | **64** | **16** | **5** | **0** | **0** | **0** | **0** | **0** |
|  | **Food** | **0** | **0** | **4** | **36** | **77** | **115** | **91** | **72** | **26** | **0** | **0** | **0** | **0** |
| **RBRC** | **Activity** | **0** | **0** | **6** | **89** | **93** | **65** | **24** | **0** | **0** | **0** | **0** | **0** | **0** |
|  | **Food** | **0** | **0** | **0** | **27** | **66** | **59** | **46** | **21** | **7** | **1** | **0** | **0** | **0** |
| **TCP** | **Activity** | **0** | **0** | **8** | **8** | **31** | **42** | **23** | **8** | **2** | **6** | **1** | **0** | **0** |
|  | **Food** | **0** | **0** | **0** | **0** | **10** | **8** | **24** | **24** | **16** | **12** | **1** | **0** | **0** |
| **HMGU** | **Activity** | **0** | **5** | **20** | **515** | **362** | **97** | **4** | **0** | **1** | **0** | **0** | **0** | **0** |
|  | **Food** | **0** | **1** | **8** | **65** | **201** | **328** | **219** | **91** | **27** | **7** | **5** | **1** | **0** |
