# Supplementary material for: High-throughput discovery of genetic determinants of circadian misalignment
Source: PLoS Genet. 2020 Jan 13;16(1):e1008577. doi: 10.1371/journal.pgen.1008577 (PMC6980734; doi:10.1371/journal.pgen.1008577)
Supplement: S5 Table — (DOCX) [file pgen.1008577.s009.docx]

**S5 Table. Effect size and p value of known mutants (visual assessment)**

|  | **Genotype** | **T_on_** | **T_ph_** | ***d_on_*** | ***p_on_*** | ***d_ph_*** | ***p_ph_*** |
| --- | --- | --- | --- | --- | --- | --- | --- |
| **Activity** | **wild type** | **12.14±0.38** | **14.14±1.35** |  |  |  |  |
|  | **PER2^S662G^** | **10.15±1.10** | **12.00±1.00** | **2.21** | **1.71E-4** | **1.90** | **7.41E-4** |
|  | ***Fbxl3^-/-^*** | **14.80±0.45** | **16.20±1.10** | **6.52** | **5.82E-7** | **1.64** | **1.85E-2** |
|  | ***Zbtb20^-/-^*** | **19.38±3.20** | **21.50±3.85** | **3.06** | **5.17E-5** | **2.47** | **3.58E-4** |
|  | **PER1^S714G^** | **10.00±1.62** | **12.00±1.62** | **1.54** | **2.46E-3** | **1.38** | **4.5E-2** |
| **Food**  **intake** | **wild type** | **11.57±0.98** | **14.00±1.15** |  |  |  |  |
|  | **PER2^S662G^** | **9.77±1.09** | **11.61±1.26** | **1.69** | **1.85E-3** | **1.93** | **6.05E-4** |
|  | ***Fbxl3^-/-^*** | **11.8±1.10** | **16.20±1.64** | **0.22** | **0.71** | **1.60** | **2.1E-2** |
|  | ***Zbtb20^-/-^*** | **10.63±** | **13.50±2.93** | **1.10** | **3.7E-2** | **0.24** | **0.35** |
|  | **PER1^S714G^** | **7.63±1.12** | **9.68±1.97** | **3.64** | **1.90E-8** | **2.39** | **1.47E-5** |

**T_on_: onset time**

**T_ph_: peak phase**

***d_on_*: effect size for onset times**

***p_on_*: p value of student’s test of wild type and known mutants’ onset times**

***d_ph_*: effect size for peak phases**

***p_ph_*: p value of student’s test of wild type and known mutant’s peak phases**
