# Supplementary material for: High-throughput discovery of genetic determinants of circadian misalignment
Source: PLoS Genet. 2020 Jan 13;16(1):e1008577. doi: 10.1371/journal.pgen.1008577 (PMC6980734; doi:10.1371/journal.pgen.1008577)
Supplement: S6 Table — (DOCX) [file pgen.1008577.s010.docx]

**S6 Table. Number of mutant lines in each center**

| **Center** | **Heterozygote (genes)** | **Homozygote (genes)** |
| --- | --- | --- |
| **WTSI** | **86** | **195** |
| **ICS** | **42** | **39** |
| **RBRC** | **9** | **20** |
| **TCP** | **67** | **59** |
| **HGMU** | **106** | **127** |
