# Supplementary material for: High-throughput discovery of genetic determinants of circadian misalignment
Source: PLoS Genet. 2020 Jan 13;16(1):e1008577. doi: 10.1371/journal.pgen.1008577 (PMC6980734; doi:10.1371/journal.pgen.1008577)
Supplement: S8 Table — (DOCX) [file pgen.1008577.s012.docx]

**S8 Table. Onset times and peak phases of mutant lines**

| **Center** | **gene ID** | **gene Symbol** | **Activity** | | **Food** **intake** | |
| --- | --- | --- | --- | --- | --- | --- |
|  |  |  | **Phase** | **Onset** | **Phase** | **Onset** |
| **WTSI** | **MGI:101835** | **Traf2** | **14.16** | **12.49** | **15.81** | **11.98** |
| **WTSI** | **MGI:101920** | **Ap2a2** | **14.54** | **12.54** | **17.02** | **13.56** |
| **WTSI** | **MGI:102848** | **Serpina3c** | **14.11** | **12.54** | **14.81** | **13.77** |
| **WTSI** | **MGI:103221** | **Dsc2** | **14.09** | **12.49** | **13.57** | **11.98** |
| **WTSI** | **MGI:103263** | **Mcf2l** | **14.44** | **12.49** | **15.28** | **12.41** |
| **WTSI** | **MGI:104510** | **Myo7a** | **18.00** | **12.49** | **17.25** | **11.98** |
| **WTSI** | **MGI:104807** | **Pls3** | **14.19** | **12.49** | **18.24** | **13.66** |
| **WTSI** | **MGI:104837** | **Amz2** | **15.01** | **12.49** | **17.56** | **13.01** |
| **WTSI** | **MGI:104848** | **Inpp1** | **14.22** | **12.49** | **16.40** | **12.92** |
| **WTSI** | **MGI:104849** | **Cd55b** | **13.95** | **12.68** | **15.07** | **12.00** |
| **WTSI** | **MGI:104967** | **Glg1** | **14.29** | **12.63** | **17.74** | **13.63** |
| **WTSI** | **MGI:106441** | **Zranb1** | **15.60** | **12.52** | **14.77** | **12.02** |
| **WTSI** | **MGI:106927** | **Aff3** | **13.83** | **12.49** | **13.77** | **12.96** |
| **WTSI** | **MGI:107403** | **Clk1** | **13.77** | **12.49** | **14.80** | **11.99** |
| **WTSI** | **MGI:107566** | **Kifap3** | **13.96** | **12.61** | **13.91** | **11.99** |
| **WTSI** | **MGI:107684** | **Plxna2** | **14.32** | **12.49** | **15.66** | **12.08** |
| **WTSI** | **MGI:107716** | **Myo10** | **13.80** | **12.49** | **15.31** | **11.97** |
| **WTSI** | **MGI:107745** | **Dctn1** | **13.82** | **12.49** | **13.77** | **11.99** |
| **WTSI** | **MGI:108048** | **Smyd5** | **14.08** | **12.51** | **14.61** | **13.06** |
| **WTSI** | **MGI:108072** | **Traf6** | **14.30** | **12.57** | **16.32** | **13.00** |
| **WTSI** | **MGI:108076** | **Frrs1** | **14.00** | **12.56** | **14.12** | **12.23** |
| **WTSI** | **MGI:109176** | **Cpt2** | **13.97** | **12.55** | **14.95** | **12.00** |
| **WTSI** | **MGI:109331** | **Nxn** | **14.54** | **12.62** | **17.17** | **12.85** |
| **WTSI** | **MGI:109380** | **Il10rb** | **14.38** | **12.53** | **16.72** | **12.99** |
| **WTSI** | **MGI:109406** | **Reg3g** | **14.11** | **12.62** | **17.10** | **12.96** |
| **WTSI** | **MGI:109567** | **Wbp5** | **14.43** | **12.49** | **16.10** | **13.00** |
| **WTSI** | **MGI:109620** | **Arvcf** | **14.54** | **12.49** | **15.62** | **12.99** |
| **WTSI** | **MGI:1096566** | **Pias2** | **14.16** | **12.60** | **16.11** | **13.20** |
| **WTSI** | **MGI:1098267** | **Ogdh** | **14.09** | **12.49** | **17.06** | **12.43** |
| **WTSI** | **MGI:1098547** | **Nhp2** | **14.10** | **12.49** | **15.08** | **11.97** |
| **WTSI** | **MGI:1098686** | **Tmc6** | **14.54** | **12.49** | **13.69** | **12.39** |
| **WTSI** | **MGI:1098826** | **Coq4** | **14.10** | **12.49** | **15.05** | **11.99** |
| **WTSI** | **MGI:1195262** | **Man2b2** | **15.14** | **12.66** | **17.53** | **12.99** |
| **WTSI** | **MGI:1202710** | **Art4** | **14.17** | **12.49** | **14.00** | **12.45** |
| **WTSI** | **MGI:1261422** | **Naga** | **13.96** | **12.54** | **15.29** | **12.98** |
| **WTSI** | **MGI:1261797** | **Cdkn2aipnl** | **15.44** | **12.57** | **16.33** | **13.05** |
| **WTSI** | **MGI:1261811** | **Myo15** | **13.76** | **12.55** | **14.81** | **11.99** |
| **WTSI** | **MGI:1261827** | **Dnmt3a** | **14.23** | **12.54** | **15.21** | **12.98** |
| **WTSI** | **MGI:1289168** | **Cd300lg** | **14.10** | **12.49** | **15.92** | **12.98** |
| **WTSI** | **MGI:1306779** | **Ccl22** | **13.72** | **12.49** | **13.38** | **12.00** |
| **WTSI** | **MGI:1312922** | **Casp12** | **14.31** | **12.49** | **13.78** | **13.25** |
| **WTSI** | **MGI:1316658** | **Cyba** | **14.28** | **12.56** | **15.81** | **12.98** |
| **WTSI** | **MGI:1316742** | **Lgals7** | **14.20** | **12.49** | **16.66** | **11.98** |
| **WTSI** | **MGI:1334448** | **Aspm** | **14.54** | **12.49** | **13.93** | **11.98** |
| **WTSI** | **MGI:1336993** | **Ap4e1** | **14.54** | **12.49** | **16.28** | **12.00** |
| **WTSI** | **MGI:1338759** | **Sec22b** | **13.85** | **12.49** | **14.03** | **12.00** |
| **WTSI** | **MGI:1341155** | **Gldc** | **14.15** | **12.57** | **14.75** | **12.95** |
| **WTSI** | **MGI:1343044** | **Rbmx** | **14.17** | **12.49** | **14.04** | **11.99** |
| **WTSI** | **MGI:1343177** | **Calcoco2** | **13.83** | **12.49** | **17.03** | **13.03** |
| **WTSI** | **MGI:1344351** | **Dlg2** | **14.18** | **12.49** | **15.85** | **11.97** |
| **WTSI** | **MGI:1345964** | **Coro1c** | **14.33** | **12.61** | **16.76** | **13.67** |
| **WTSI** | **MGI:1346328** | **Def6** | **13.87** | **12.49** | **16.19** | **11.98** |
| **WTSI** | **MGI:1346871** | **Map2k7** | **14.14** | **12.49** | **15.37** | **11.99** |
| **WTSI** | **MGI:1346872** | **Map3k1** | **14.46** | **12.49** | **16.59** | **12.97** |
| **WTSI** | **MGI:1347062** | **Creb3l1** | **14.02** | **12.49** | **14.00** | **11.99** |
| **WTSI** | **MGI:1349457** | **Serinc3** | **14.10** | **12.52** | **13.93** | **11.96** |
| **WTSI** | **MGI:1351345** | **Grm8** | **14.54** | **12.49** | **16.11** | **13.89** |
| **WTSI** | **MGI:1351869** | **Pdxk** | **14.00** | **12.49** | **14.97** | **11.98** |
| **WTSI** | **MGI:1352750** | **Cabp1** | **14.03** | **12.49** | **15.15** | **13.00** |
| **WTSI** | **MGI:1353665** | **Usp21** | **14.03** | **12.67** | **17.55** | **12.97** |
| **WTSI** | **MGI:1354944** | **Orc3** | **14.01** | **12.49** | **13.20** | **11.98** |
| **WTSI** | **MGI:1859212** | **Irf7** | **14.17** | **12.65** | **14.34** | **12.97** |
| **WTSI** | **MGI:1859328** | **Cpsf3** | **14.39** | **12.49** | **15.37** | **11.98** |
| **WTSI** | **MGI:1860086** | **Crlf3** | **13.69** | **12.49** | **12.62** | **11.98** |
| **WTSI** | **MGI:1860493** | **Arhgef7** | **14.99** | **12.53** | **15.47** | **11.97** |
| **WTSI** | **MGI:1861457** | **Dynll1** | **13.64** | **12.49** | **14.72** | **11.99** |
| **WTSI** | **MGI:1861607** | **Gbf1** | **14.30** | **12.51** | **16.35** | **12.98** |
| **WTSI** | **MGI:1890773** | **Actn4** | **13.76** | **12.55** | **15.18** | **12.99** |
| **WTSI** | **MGI:1891410** | **Pacsin3** | **15.37** | **12.49** | **16.24** | **12.98** |
| **WTSI** | **MGI:1913411** | **Pop4** | **13.93** | **12.57** | **15.17** | **12.31** |
| **WTSI** | **MGI:1913435** | **1110037F02Rik** | **14.59** | **12.57** | **16.14** | **13.08** |
| **WTSI** | **MGI:1913452** | **1110059G10Rik** | **15.45** | **12.54** | **17.18** | **13.00** |
| **WTSI** | **MGI:1913486** | **1500011B03Rik** | **13.76** | **12.49** | **13.81** | **11.98** |
| **WTSI** | **MGI:1913560** | **Dpy30** | **14.17** | **12.49** | **15.82** | **12.99** |
| **WTSI** | **MGI:1913649** | **Tsfm** | **15.27** | **12.72** | **15.43** | **11.98** |
| **WTSI** | **MGI:1913840** | **Farsa** | **13.76** | **12.49** | **13.57** | **11.99** |
| **WTSI** | **MGI:1913955** | **Dnase1l2** | **14.20** | **12.53** | **16.35** | **12.99** |
| **WTSI** | **MGI:1914050** | **Trp53rkb** | **14.29** | **12.51** | **15.86** | **11.97** |
| **WTSI** | **MGI:1914269** | **Actr6** | **14.20** | **12.49** | **14.89** | **12.01** |
| **WTSI** | **MGI:1914318** | **Dynlrb1** | **13.90** | **12.49** | **16.24** | **11.98** |
| **WTSI** | **MGI:1914338** | **Cand2** | **14.29** | **12.49** | **13.79** | **12.94** |
| **WTSI** | **MGI:1914361** | **Naaa** | **13.93** | **12.49** | **16.62** | **13.01** |
| **WTSI** | **MGI:1914450** | **Ccdc77** | **13.92** | **12.49** | **14.42** | **11.98** |
| **WTSI** | **MGI:1914681** | **4933402N03Rik** | **13.96** | **12.49** | **16.19** | **12.21** |
| **WTSI** | **MGI:1914804** | **Slc25a30** | **14.16** | **12.49** | **16.37** | **12.02** |
| **WTSI** | **MGI:1914824** | **Alg13** | **13.69** | **12.54** | **15.13** | **11.98** |
| **WTSI** | **MGI:1914933** | **Pbdc1** | **13.96** | **12.49** | **14.28** | **11.99** |
| **WTSI** | **MGI:1914978** | **Dph2** | **13.98** | **12.54** | **17.43** | **13.19** |
| **WTSI** | **MGI:1915098** | **Ddx55** | **13.80** | **12.49** | **14.74** | **11.99** |
| **WTSI** | **MGI:1915367** | **Apool** | **13.69** | **12.49** | **13.72** | **12.99** |
| **WTSI** | **MGI:1915425** | **4930591A17Rik** | **14.30** | **12.49** | **17.06** | **13.35** |
| **WTSI** | **MGI:1915459** | **Rnaseh2c** | **13.87** | **12.52** | **15.80** | **11.98** |
| **WTSI** | **MGI:1915500** | **Fam96a** | **13.75** | **12.53** | **13.77** | **11.99** |
| **WTSI** | **MGI:1915509** | **Ift80** | **14.00** | **12.49** | **15.64** | **12.68** |
| **WTSI** | **MGI:1915549** | **Vps53** | **14.54** | **12.49** | **16.27** | **11.98** |
| **WTSI** | **MGI:1915761** | **Dcdc2c** | **14.03** | **12.52** | **15.29** | **12.00** |
| **WTSI** | **MGI:1916003** | **Mybphl** | **14.54** | **12.49** | **15.88** | **12.79** |
| **WTSI** | **MGI:1916205** | **Srrm4** | **14.44** | **12.49** | **15.24** | **11.98** |
| **WTSI** | **MGI:1916222** | **Tatdn3** | **14.30** | **12.49** | **14.92** | **11.98** |
| **WTSI** | **MGI:1916264** | **Tnik** | **13.87** | **12.55** | **16.10** | **11.97** |
| **WTSI** | **MGI:1916599** | **1700008O03Rik** | **14.22** | **12.61** | **17.29** | **12.31** |
| **WTSI** | **MGI:1916706** | **Commd10** | **14.11** | **12.58** | **16.09** | **12.99** |
| **WTSI** | **MGI:1916865** | **Rab15** | **14.37** | **12.81** | **16.83** | **13.00** |
| **WTSI** | **MGI:1916998** | **Aldh16a1** | **14.16** | **12.49** | **14.94** | **11.98** |
| **WTSI** | **MGI:1917475** | **Ppil3** | **13.90** | **12.57** | **14.99** | **11.98** |
| **WTSI** | **MGI:1917672** | **Ints2** | **13.90** | **12.49** | **15.30** | **11.98** |
| **WTSI** | **MGI:1917682** | **Rufy2** | **13.77** | **12.49** | **16.07** | **12.84** |
| **WTSI** | **MGI:1917708** | **2610318N02Rik** | **13.98** | **12.67** | **15.85** | **12.02** |
| **WTSI** | **MGI:1917946** | **3830417A13Rik** | **14.50** | **12.71** | **16.68** | **13.00** |
| **WTSI** | **MGI:1918035** | **Dennd1c** | **14.12** | **12.49** | **14.01** | **11.99** |
| **WTSI** | **MGI:1918358** | **Ccdc122** | **13.96** | **12.49** | **15.48** | **11.98** |
| **WTSI** | **MGI:1918614** | **Jmjd1c** | **13.99** | **12.50** | **15.52** | **11.99** |
| **WTSI** | **MGI:1918662** | **Dhrs2** | **14.12** | **12.49** | **16.12** | **11.98** |
| **WTSI** | **MGI:1918876** | **Gimap6** | **15.40** | **12.49** | **15.82** | **12.99** |
| **WTSI** | **MGI:1918970** | **Osbpl3** | **14.54** | **12.62** | **16.55** | **12.73** |
| **WTSI** | **MGI:1919073** | **3300002A11Rik** | **14.86** | **12.52** | **16.92** | **12.47** |
| **WTSI** | **MGI:1919268** | **Fundc1** | **14.23** | **12.49** | **14.95** | **12.98** |
| **WTSI** | **MGI:1919288** | **Klhl21** | **15.52** | **12.54** | **17.53** | **12.92** |
| **WTSI** | **MGI:1919305** | **Slc38a10** | **15.07** | **12.49** | **15.96** | **12.98** |
| **WTSI** | **MGI:1919338** | **Ush1c** | **16.79** | **14.18** | **15.99** | **12.99** |
| **WTSI** | **MGI:1919374** | **Seh1l** | **14.30** | **12.64** | **15.23** | **12.99** |
| **WTSI** | **MGI:1919419** | **Trim29** | **13.94** | **12.49** | **15.09** | **11.98** |
| **WTSI** | **MGI:1919918** | **Skida1** | **13.88** | **12.53** | **13.32** | **12.02** |
| **WTSI** | **MGI:1920040** | **Ssbp1** | **15.56** | **12.61** | **18.49** | **13.26** |
| **WTSI** | **MGI:1920223** | **Fbxo47** | **13.86** | **12.49** | **13.50** | **12.00** |
| **WTSI** | **MGI:1920393** | **Wdr37** | **14.80** | **12.54** | **15.89** | **12.97** |
| **WTSI** | **MGI:1920432** | **Pear1** | **15.65** | **12.50** | **17.69** | **13.99** |
| **WTSI** | **MGI:1920563** | **Rpgrip1l** | **13.73** | **12.49** | **14.58** | **11.99** |
| **WTSI** | **MGI:1920597** | **Cldn34b2** | **14.17** | **12.61** | **16.77** | **12.92** |
| **WTSI** | **MGI:1920864** | **Rhox13** | **14.54** | **12.49** | **16.64** | **12.99** |
| **WTSI** | **MGI:1920893** | **1700123O20Rik** | **14.54** | **12.59** | **16.32** | **13.06** |
| **WTSI** | **MGI:1921082** | **Klk9** | **14.74** | **12.62** | **17.14** | **11.97** |
| **WTSI** | **MGI:1921138** | **Ppp1r42** | **15.28** | **12.49** | **15.93** | **13.00** |
| **WTSI** | **MGI:1921258** | **Arsg** | **14.54** | **12.49** | **16.61** | **12.44** |
| **WTSI** | **MGI:1921354** | **Abcb6** | **15.26** | **12.58** | **16.59** | **12.98** |
| **WTSI** | **MGI:1921501** | **Ankrd9** | **14.02** | **12.49** | **16.48** | **11.98** |
| **WTSI** | **MGI:1921615** | **Lonrf3** | **14.16** | **12.52** | **14.96** | **12.07** |
| **WTSI** | **MGI:1921637** | **4932438H23Rik** | **14.87** | **12.49** | **15.16** | **11.98** |
| **WTSI** | **MGI:1921660** | **Ttll11** | **13.91** | **12.49** | **14.52** | **11.98** |
| **WTSI** | **MGI:1921662** | **Gle1** | **13.89** | **12.49** | **14.40** | **13.02** |
| **WTSI** | **MGI:1921690** | **Cmip** | **14.17** | **12.63** | **15.59** | **12.81** |
| **WTSI** | **MGI:1921713** | **Exoc3l2** | **14.89** | **12.49** | **17.12** | **12.96** |
| **WTSI** | **MGI:1921765** | **Cdkal1** | **13.83** | **12.49** | **15.23** | **12.98** |
| **WTSI** | **MGI:1922032** | **Glt8d2** | **14.54** | **12.51** | **14.79** | **11.98** |
| **WTSI** | **MGI:1922516** | **Tomm20l** | **13.78** | **12.67** | **14.86** | **11.98** |
| **WTSI** | **MGI:1922670** | **Secisbp2** | **14.54** | **12.49** | **13.75** | **12.97** |
| **WTSI** | **MGI:1923549** | **Erp44** | **14.17** | **12.52** | **15.59** | **12.03** |
| **WTSI** | **MGI:1923691** | **Daam2** | **14.16** | **12.54** | **17.44** | **11.98** |
| **WTSI** | **MGI:1923707** | **Ccdc134** | **14.48** | **12.49** | **16.91** | **13.03** |
| **WTSI** | **MGI:1924015** | **Mlec** | **14.67** | **12.64** | **17.15** | **12.44** |
| **WTSI** | **MGI:1924086** | **Polr3f** | **14.17** | **12.49** | **15.77** | **12.97** |
| **WTSI** | **MGI:1924232** | **3110035E14Rik** | **13.97** | **12.49** | **14.35** | **11.98** |
| **WTSI** | **MGI:1924301** | **Os9** | **14.04** | **12.49** | **15.47** | **12.97** |
| **WTSI** | **MGI:1924781** | **Anks1b** | **15.59** | **12.60** | **16.44** | **12.98** |
| **WTSI** | **MGI:1924817** | **Tram2** | **14.54** | **12.49** | **17.19** | **12.94** |
| **WTSI** | **MGI:1924919** | **Arhgef38** | **14.99** | **12.64** | **17.23** | **13.01** |
| **WTSI** | **MGI:1924971** | **Mrps5** | **14.54** | **12.53** | **16.15** | **12.99** |
| **WTSI** | **MGI:1925201** | **Cyp20a1** | **14.54** | **12.49** | **16.48** | **13.09** |
| **WTSI** | **MGI:1925537** | **Rbsn** | **13.76** | **12.49** | **14.24** | **11.98** |
| **WTSI** | **MGI:1926074** | **Ubash3a** | **14.17** | **12.55** | **16.13** | **12.99** |
| **WTSI** | **MGI:1926116** | **Fam175b** | **14.54** | **12.64** | **16.87** | **13.00** |
| **WTSI** | **MGI:1926232** | **Srsf7** | **14.54** | **12.49** | **15.54** | **12.07** |
| **WTSI** | **MGI:1927243** | **Rala** | **14.54** | **12.49** | **15.11** | **11.98** |
| **WTSI** | **MGI:1927244** | **Ralb** | **14.17** | **12.49** | **13.18** | **12.82** |
| **WTSI** | **MGI:1927596** | **Gprc5b** | **15.26** | **12.74** | **16.40** | **12.98** |
| **WTSI** | **MGI:1927665** | **Sirt3** | **13.57** | **12.49** | **12.25** | **12.16** |
| **WTSI** | **MGI:1928895** | **Isg20** | **14.83** | **12.62** | **16.11** | **12.99** |
| **WTSI** | **MGI:1929596** | **Prrg2** | **14.54** | **12.52** | **15.46** | **12.99** |
| **WTSI** | **MGI:1931148** | **Nme4** | **14.08** | **12.49** | **15.38** | **13.46** |
| **WTSI** | **MGI:1931466** | **Selenok** | **14.33** | **12.56** | **15.84** | **11.99** |
| **WTSI** | **MGI:1933131** | **Kcnip4** | **14.00** | **12.49** | **13.45** | **11.99** |
| **WTSI** | **MGI:1933212** | **Ngrn** | **14.20** | **12.49** | **16.29** | **12.99** |
| **WTSI** | **MGI:2135601** | **Slc1a4** | **14.34** | **12.49** | **17.50** | **12.25** |
| **WTSI** | **MGI:2137679** | **Sfxn3** | **14.18** | **12.49** | **13.88** | **11.98** |
| **WTSI** | **MGI:2138198** | **D630023F18Rik** | **13.84** | **12.49** | **15.05** | **11.99** |
| **WTSI** | **MGI:2139054** | **Gpr107** | **13.92** | **12.56** | **14.16** | **11.97** |
| **WTSI** | **MGI:2139220** | **Tm9sf4** | **13.92** | **12.49** | **14.46** | **11.98** |
| **WTSI** | **MGI:2139365** | **Abtb2** | **15.23** | **12.64** | **17.73** | **12.98** |
| **WTSI** | **MGI:2140680** | **A430005L14Rik** | **14.93** | **12.69** | **18.51** | **13.02** |
| **WTSI** | **MGI:2142624** | **Tmem189** | **14.94** | **12.49** | **16.51** | **11.98** |
| **WTSI** | **MGI:2142888** | **Cmtm4** | **14.05** | **12.49** | **14.81** | **12.99** |
| **WTSI** | **MGI:2144506** | **Rundc1** | **14.02** | **12.49** | **14.58** | **11.96** |
| **WTSI** | **MGI:2144585** | **Slc16a6** | **14.18** | **12.49** | **15.09** | **11.98** |
| **WTSI** | **MGI:2146443** | **Xxylt1** | **13.82** | **12.49** | **15.66** | **11.97** |
| **WTSI** | **MGI:2148491** | **Acaa1a** | **13.96** | **12.49** | **14.37** | **12.41** |
| **WTSI** | **MGI:2150302** | **Ddhd1** | **14.20** | **12.49** | **14.88** | **12.97** |
| **WTSI** | **MGI:2152450** | **Usp3** | **15.04** | **12.68** | **18.38** | **13.61** |
| **WTSI** | **MGI:2153044** | **Elmo1** | **13.73** | **12.49** | **15.81** | **11.99** |
| **WTSI** | **MGI:2154278** | **Ankrd6** | **13.82** | **12.55** | **14.63** | **11.98** |
| **WTSI** | **MGI:2157522** | **Dppa1** | **13.96** | **12.49** | **13.93** | **11.98** |
| **WTSI** | **MGI:2159407** | **Zcchc14** | **14.30** | **12.49** | **15.95** | **12.46** |
| **WTSI** | **MGI:2179326** | **Oxr1** | **13.95** | **12.49** | **14.23** | **12.00** |
| **WTSI** | **MGI:2180917** | **Pth2r** | **14.03** | **12.49** | **14.10** | **11.97** |
| **WTSI** | **MGI:2181068** | **Klf17** | **13.81** | **12.52** | **16.45** | **12.96** |
| **WTSI** | **MGI:2181962** | **Acer1** | **14.97** | **12.53** | **17.76** | **12.99** |
| **WTSI** | **MGI:2182357** | **Ropn1l** | **14.70** | **12.49** | **16.16** | **12.99** |
| **WTSI** | **MGI:2183438** | **Ell2** | **13.94** | **12.49** | **13.76** | **11.99** |
| **WTSI** | **MGI:2384312** | **Usp11** | **14.13** | **12.49** | **16.87** | **11.97** |
| **WTSI** | **MGI:2384790** | **Fanci** | **13.89** | **12.57** | **17.04** | **11.98** |
| **WTSI** | **MGI:2384806** | **Metrnl** | **14.51** | **12.50** | **17.38** | **11.98** |
| **WTSI** | **MGI:2384936** | **Spns2** | **13.79** | **12.49** | **14.14** | **11.97** |
| **WTSI** | **MGI:2385160** | **Bpifb5** | **14.29** | **12.49** | **16.09** | **13.00** |
| **WTSI** | **MGI:2385184** | **Ahcyl1** | **13.77** | **12.49** | **14.23** | **11.98** |
| **WTSI** | **MGI:2385206** | **Pabpc4** | **14.20** | **12.49** | **15.82** | **13.06** |
| **WTSI** | **MGI:2386851** | **Rasgrp4** | **13.98** | **12.49** | **15.30** | **11.98** |
| **WTSI** | **MGI:2387643** | **B9d2** | **16.11** | **12.49** | **13.71** | **11.98** |
| **WTSI** | **MGI:2429554** | **Pdzd3** | **14.16** | **12.49** | **16.66** | **12.64** |
| **WTSI** | **MGI:2442040** | **G3bp2** | **13.94** | **12.49** | **13.01** | **11.98** |
| **WTSI** | **MGI:2442056** | **Pld5** | **15.28** | **12.64** | **15.81** | **12.98** |
| **WTSI** | **MGI:2442201** | **Adap1** | **14.36** | **12.49** | **15.33** | **11.98** |
| **WTSI** | **MGI:2442220** | **Zfp182** | **14.54** | **12.49** | **13.69** | **11.99** |
| **WTSI** | **MGI:2442510** | **Dars2** | **14.54** | **12.49** | **15.85** | **11.98** |
| **WTSI** | **MGI:2443388** | **Kdm7a** | **14.10** | **12.49** | **15.37** | **12.19** |
| **WTSI** | **MGI:2443418** | **Arhgap22** | **13.96** | **12.49** | **15.69** | **12.10** |
| **WTSI** | **MGI:2443470** | **Mrm1** | **14.31** | **12.56** | **15.17** | **11.98** |
| **WTSI** | **MGI:2443584** | **L3mbtl2** | **14.79** | **12.49** | **18.13** | **13.17** |
| **WTSI** | **MGI:2443686** | **Rtbdn** | **13.85** | **12.54** | **15.08** | **11.98** |
| **WTSI** | **MGI:2443881** | **Rasal2** | **14.41** | **12.49** | **16.56** | **12.37** |
| **WTSI** | **MGI:2444067** | **Pqlc3** | **14.29** | **12.49** | **16.90** | **13.04** |
| **WTSI** | **MGI:2444110** | **Sgms1** | **15.04** | **12.49** | **14.98** | **12.97** |
| **WTSI** | **MGI:2444531** | **Ralgapb** | **13.84** | **12.49** | **13.24** | **11.98** |
| **WTSI** | **MGI:2444708** | **Zfp719** | **13.86** | **12.49** | **13.32** | **11.97** |
| **WTSI** | **MGI:2446215** | **Sh2d5** | **14.32** | **12.49** | **16.63** | **12.99** |
| **WTSI** | **MGI:2446237** | **Vps33b** | **14.45** | **12.49** | **15.79** | **11.98** |
| **WTSI** | **MGI:2446249** | **Edc4** | **15.01** | **12.58** | **18.05** | **13.27** |
| **WTSI** | **MGI:2446634** | **Ago3** | **14.00** | **12.71** | **16.69** | **13.36** |
| **WTSI** | **MGI:2447165** | **Cmtm6** | **14.17** | **12.49** | **14.42** | **11.98** |
| **WTSI** | **MGI:2447812** | **Dennd1b** | **14.60** | **12.49** | **16.49** | **12.99** |
| **WTSI** | **MGI:2448554** | **Nbeal2** | **13.80** | **12.52** | **14.32** | **11.97** |
| **WTSI** | **MGI:2448556** | **Stard8** | **14.03** | **12.49** | **15.98** | **11.98** |
| **WTSI** | **MGI:2449771** | **Cyp2r1** | **14.17** | **12.58** | **16.80** | **12.81** |
| **WTSI** | **MGI:2664357** | **Dsg1b** | **14.17** | **12.49** | **13.54** | **11.99** |
| **WTSI** | **MGI:2670981** | **Kcnv2** | **14.13** | **12.55** | **15.78** | **11.98** |
| **WTSI** | **MGI:2673872** | **Syt16** | **13.97** | **12.49** | **15.67** | **11.99** |
| **WTSI** | **MGI:2679260** | **Crb2** | **14.54** | **12.49** | **15.43** | **12.98** |
| **WTSI** | **MGI:2679270** | **Zkscan17** | **14.02** | **12.49** | **15.60** | **11.99** |
| **WTSI** | **MGI:2679719** | **Armc7** | **14.14** | **12.53** | **16.45** | **12.17** |
| **WTSI** | **MGI:2684992** | **Zfyve28** | **14.95** | **12.49** | **17.28** | **13.01** |
| **WTSI** | **MGI:2685494** | **Gm648** | **14.17** | **12.49** | **14.81** | **11.98** |
| **WTSI** | **MGI:2685541** | **Wdtc1** | **15.03** | **12.52** | **17.17** | **12.00** |
| **WTSI** | **MGI:2685874** | **Ccdc24** | **14.19** | **12.67** | **16.32** | **13.62** |
| **WTSI** | **MGI:2687005** | **Leprot** | **14.54** | **12.58** | **12.86** | **12.29** |
| **WTSI** | **MGI:2687284** | **Pfkfb4** | **14.25** | **12.49** | **17.39** | **12.97** |
| **WTSI** | **MGI:3028035** | **A830019P07Rik** | **13.98** | **12.57** | **14.98** | **11.98** |
| **WTSI** | **MGI:3036236** | **Tceal5** | **14.12** | **12.49** | **14.45** | **12.53** |
| **WTSI** | **MGI:3039628** | **Rsad1** | **14.54** | **12.62** | **15.70** | **13.54** |
| **WTSI** | **MGI:3040700** | **Zscan10** | **13.94** | **12.49** | **15.83** | **12.77** |
| **WTSI** | **MGI:3045338** | **Mettl24** | **13.68** | **12.49** | **15.40** | **11.98** |
| **WTSI** | **MGI:3046173** | **Nup85** | **14.11** | **12.59** | **17.22** | **12.99** |
| **WTSI** | **MGI:3512628** | **Cbx6** | **14.96** | **12.55** | **16.84** | **13.11** |
| **WTSI** | **MGI:3583955** | **BC089597** | **13.95** | **12.49** | **16.19** | **13.00** |
| **WTSI** | **MGI:3584533** | **Spink10** | **14.17** | **12.49** | **16.02** | **13.17** |
| **WTSI** | **MGI:3588198** | **Cdh19** | **14.09** | **12.48** | **15.23** | **11.99** |
| **WTSI** | **MGI:3588225** | **Ccdc160** | **14.17** | **12.49** | **15.01** | **12.04** |
| **WTSI** | **MGI:3603030** | **Nacad** | **14.57** | **12.49** | **14.34** | **12.99** |
| **WTSI** | **MGI:3609239** | **Mrap2** | **14.95** | **12.49** | **15.75** | **13.16** |
| **WTSI** | **MGI:3612340** | **Ehbp1l1** | **15.08** | **12.49** | **18.15** | **12.94** |
| **WTSI** | **MGI:3646662** | **Fbxw26** | **14.07** | **12.49** | **15.17** | **12.01** |
| **WTSI** | **MGI:3650473** | **Gm13547** | **14.81** | **12.49** | **14.55** | **13.01** |
| **WTSI** | **MGI:3650508** | **Cnbd1** | **13.74** | **12.49** | **15.53** | **11.98** |
| **WTSI** | **MGI:3650906** | **Zfp616** | **14.06** | **12.49** | **14.86** | **12.20** |
| **WTSI** | **MGI:3710397** | **Tmem254b** | **13.69** | **12.53** | **16.42** | **11.98** |
| **WTSI** | **MGI:3712553** | **Gm13125** | **14.39** | **12.54** | **15.97** | **12.14** |
| **WTSI** | **MGI:88135** | **Tff1** | **13.95** | **12.49** | **14.91** | **11.97** |
| **WTSI** | **MGI:88609** | **Cyp3a11** | **15.25** | **12.65** | **14.92** | **12.99** |
| **WTSI** | **MGI:894318** | **Cdk14** | **14.06** | **12.49** | **14.66** | **11.98** |
| **WTSI** | **MGI:96704** | **Krt7** | **13.95** | **12.49** | **14.10** | **11.99** |
| **WTSI** | **MGI:97401** | **Ocm** | **14.00** | **12.49** | **14.53** | **13.66** |
| **WTSI** | **MGI:97429** | **Oas1g** | **13.97** | **12.49** | **15.64** | **12.04** |
| **WTSI** | **MGI:97549** | **Pfn1** | **14.20** | **12.49** | **16.36** | **11.97** |
| **WTSI** | **MGI:97799** | **Pth** | **14.05** | **12.49** | **15.95** | **12.98** |
| **WTSI** | **MGI:97800** | **Pthlh** | **15.02** | **12.49** | **16.75** | **13.02** |
| **WTSI** | **MGI:98227** | **Sag** | **14.68** | **12.59** | **16.48** | **12.99** |
| **WTSI** | **MGI:98446** | **Med22** | **14.54** | **12.49** | **16.64** | **13.01** |
| **WTSI** | **MGI:98506** | **Tcf4** | **13.83** | **12.52** | **15.87** | **12.99** |
| **WTSI** | **MGI:98640** | **Tcte1** | **13.96** | **12.54** | **14.29** | **12.97** |
| **WTSI** | **MGI:98931** | **Ezr** | **14.08** | **12.49** | **15.37** | **13.01** |
| **WTSI** | **MGI:98955** | **Wnt3** | **14.16** | **12.49** | **16.16** | **11.98** |
| **WTSI** | **MGI:98970** | **Xbp1** | **14.17** | **12.49** | **14.34** | **13.00** |
| **WTSI** | **MGI:99176** | **Zscan2** | **14.07** | **12.49** | **15.37** | **13.03** |
| **WTSI** | **MGI:99667** | **Syt1** | **14.37** | **12.62** | **15.87** | **12.59** |
| **ICS** | **MGI:101910** | **F2rl1** | **13.48** | **11.96** | **14.57** | **11.12** |
| **ICS** | **MGI:103292** | **Rab19** | **14.81** | **11.95** | **15.26** | **11.66** |
| **ICS** | **MGI:105084** | **Satb1** | **14.32** | **11.95** | **14.46** | **11.09** |
| **ICS** | **MGI:106441** | **Zranb1** | **12.62** | **11.03** | **14.59** | **11.08** |
| **ICS** | **MGI:107563** | **Nab2** | **14.57** | **12.80** | **16.98** | **11.91** |
| **ICS** | **MGI:108017** | **Laptm4a** | **12.63** | **11.05** | **15.26** | **11.35** |
| **ICS** | **MGI:109166** | **Ncs1** | **13.49** | **12.10** | **15.27** | **11.48** |
| **ICS** | **MGI:109331** | **Nxn** | **14.83** | **11.92** | **13.97** | **12.12** |
| **ICS** | **MGI:109562** | **Ackr3** | **14.35** | **12.00** | **15.59** | **12.02** |
| **ICS** | **MGI:1101059** | **Tnfrsf9** | **14.08** | **11.89** | **16.13** | **11.75** |
| **ICS** | **MGI:1194495** | **Fpr3** | **14.05** | **11.05** | **15.02** | **11.89** |
| **ICS** | **MGI:1196224** | **Cdk8** | **13.72** | **11.97** | **15.91** | **12.03** |
| **ICS** | **MGI:1276575** | **Ptdss1** | **15.92** | **12.11** | **14.89** | **11.81** |
| **ICS** | **MGI:1289155** | **Trmt1** | **14.15** | **11.97** | **15.44** | **11.82** |
| **ICS** | **MGI:1289298** | **Knstrn** | **13.89** | **11.94** | **14.47** | **11.79** |
| **ICS** | **MGI:1298232** | **Gzmk** | **14.63** | **12.11** | **13.65** | **10.97** |
| **ICS** | **MGI:1309468** | **Casq1** | **14.91** | **11.81** | **15.20** | **11.98** |
| **ICS** | **MGI:1336880** | **Eftud2** | **14.74** | **11.92** | **13.76** | **12.09** |
| **ICS** | **MGI:1336993** | **Ap4e1** | **12.84** | **12.00** | **16.81** | **11.99** |
| **ICS** | **MGI:1340806** | **Parp1** | **13.77** | **11.99** | **15.02** | **11.75** |
| **ICS** | **MGI:1347355** | **Slc7a11** | **13.06** | **11.05** | **14.37** | **11.91** |
| **ICS** | **MGI:1859162** | **Rnf10** | **13.11** | **11.98** | **17.79** | **11.74** |
| **ICS** | **MGI:1859217** | **Dnal4** | **13.47** | **11.05** | **14.49** | **11.46** |
| **ICS** | **MGI:1913576** | **Dnajc5b** | **14.76** | **11.98** | **14.99** | **11.84** |
| **ICS** | **MGI:1913955** | **Dnase1l2** | **14.85** | **11.88** | **15.93** | **11.56** |
| **ICS** | **MGI:1914430** | **Yipf5** | **14.08** | **12.76** | **13.64** | **11.36** |
| **ICS** | **MGI:1914982** | **Iah1** | **13.92** | **11.91** | **14.66** | **11.82** |
| **ICS** | **MGI:1915107** | **Ppp6c** | **13.76** | **11.73** | **15.69** | **11.59** |
| **ICS** | **MGI:1915271** | **Bphl** | **14.89** | **11.94** | **14.56** | **12.15** |
| **ICS** | **MGI:1915299** | **Tgif2** | **12.12** | **11.05** | **14.73** | **11.15** |
| **ICS** | **MGI:1915392** | **Ino80** | **13.98** | **11.96** | **13.13** | **11.91** |
| **ICS** | **MGI:1917890** | **Dcp2** | **12.84** | **11.80** | **13.37** | **11.00** |
| **ICS** | **MGI:1918180** | **Nol8** | **13.74** | **11.05** | **15.79** | **11.78** |
| **ICS** | **MGI:1919352** | **Dusp11** | **13.38** | **11.77** | **16.96** | **12.20** |
| **ICS** | **MGI:1919792** | **Pgam5** | **14.43** | **11.05** | **15.68** | **11.57** |
| **ICS** | **MGI:1920420** | **Rwdd3** | **14.41** | **11.88** | **15.86** | **11.31** |
| **ICS** | **MGI:1921084** | **Atp6v1d** | **14.23** | **11.77** | **13.45** | **11.67** |
| **ICS** | **MGI:1921256** | **Dnm1l** | **13.68** | **11.05** | **15.65** | **10.97** |
| **ICS** | **MGI:1921489** | **Iqce** | **13.85** | **11.95** | **16.57** | **11.84** |
| **ICS** | **MGI:1922647** | **Arhgap24** | **14.35** | **11.90** | **15.51** | **11.64** |
| **ICS** | **MGI:1922863** | **Med25** | **14.49** | **11.91** | **15.30** | **10.97** |
| **ICS** | **MGI:1923930** | **5330417C22Rik** | **12.46** | **11.82** | **15.74** | **11.25** |
| **ICS** | **MGI:1924337** | **Ankrd11** | **13.05** | **11.92** | **14.08** | **11.51** |
| **ICS** | **MGI:1924408** | **Coq6** | **13.97** | **11.88** | **15.52** | **11.20** |
| **ICS** | **MGI:1926224** | **Rbfox1** | **13.48** | **11.80** | **16.47** | **11.52** |
| **ICS** | **MGI:1931838** | **Dbn1** | **14.04** | **11.05** | **14.89** | **11.97** |
| **ICS** | **MGI:1932411** | **Tmem108** | **13.41** | **11.95** | **14.22** | **11.17** |
| **ICS** | **MGI:1933199** | **Setbp1** | **13.45** | **11.71** | **14.20** | **11.66** |
| **ICS** | **MGI:2138934** | **Mbd5** | **13.47** | **11.74** | **13.76** | **11.06** |
| **ICS** | **MGI:2147616** | **Otub1** | **12.08** | **11.05** | **13.52** | **11.19** |
| **ICS** | **MGI:2182607** | **Sdsl** | **14.51** | **12.01** | **14.67** | **11.59** |
| **ICS** | **MGI:2384561** | **Nae1** | **13.49** | **11.91** | **16.30** | **11.82** |
| **ICS** | **MGI:2384986** | **Rnf144b** | **13.34** | **11.64** | **13.72** | **11.39** |
| **ICS** | **MGI:2385088** | **Smc5** | **15.30** | **11.96** | **14.62** | **11.58** |
| **ICS** | **MGI:2385295** | **Sez6l2** | **14.45** | **12.08** | **14.40** | **12.02** |
| **ICS** | **MGI:2387609** | **Tmem63b** | **13.77** | **11.94** | **15.42** | **11.40** |
| **ICS** | **MGI:2387617** | **Obp2a** | **13.75** | **11.95** | **15.66** | **11.73** |
| **ICS** | **MGI:2443480** | **Setx** | **14.08** | **11.76** | **13.12** | **11.98** |
| **ICS** | **MGI:2444103** | **Ankrd27** | **14.43** | **11.92** | **14.74** | **11.60** |
| **ICS** | **MGI:2652819** | **Baiap2l2** | **14.39** | **11.94** | **14.85** | **12.28** |
| **ICS** | **MGI:2682952** | **Prdm10** | **15.19** | **11.95** | **15.62** | **11.79** |
| **ICS** | **MGI:2683857** | **Klhl29** | **14.23** | **12.00** | **14.87** | **11.93** |
| **ICS** | **MGI:2685233** | **Ptchd1** | **14.01** | **12.00** | **16.94** | **12.20** |
| **ICS** | **MGI:2685431** | **Carmil2** | **12.98** | **11.90** | **15.13** | **11.40** |
| **ICS** | **MGI:3039607** | **Pigu** | **14.45** | **11.94** | **14.50** | **11.97** |
| **ICS** | **MGI:3045314** | **4933430I17Rik** | **14.17** | **11.91** | **14.69** | **11.35** |
| **ICS** | **MGI:3617850** | **Pnpla1** | **14.64** | **11.86** | **16.00** | **11.77** |
| **ICS** | **MGI:3651534** | **Gm12258** | **14.10** | **12.12** | **14.01** | **11.69** |
| **ICS** | **MGI:88059** | **App** | **14.45** | **11.95** | **15.19** | **11.74** |
| **ICS** | **MGI:88562** | **Ctsd** | **12.93** | **11.05** | **15.29** | **11.21** |
| **ICS** | **MGI:893575** | **Plscr1** | **14.29** | **11.82** | **15.70** | **11.68** |
| **ICS** | **MGI:95281** | **Ect2** | **13.91** | **11.88** | **15.31** | **11.02** |
| **ICS** | **MGI:96705** | **Krt8** | **14.07** | **11.95** | **15.49** | **11.93** |
| **ICS** | **MGI:96794** | **Lmna** | **13.40** | **12.00** | **14.65** | **11.96** |
| **ICS** | **MGI:97011** | **Mmp9** | **13.11** | **11.80** | **14.31** | **11.62** |
| **ICS** | **MGI:97180** | **Mapt** | **14.16** | **11.97** | **14.14** | **11.79** |
| **ICS** | **MGI:97381** | **Ntf5** | **14.19** | **11.94** | **17.13** | **12.20** |
| **ICS** | **MGI:97488** | **Pax4** | **13.95** | **11.99** | **13.00** | **11.90** |
| **ICS** | **MGI:97511** | **Pcsk1** | **14.40** | **11.05** | **16.76** | **11.59** |
| **ICS** | **MGI:97847** | **Raf1** | **13.77** | **11.78** | **14.55** | **11.59** |
| **ICS** | **MGI:98742** | **Thra** | **13.44** | **12.04** | **15.40** | **12.12** |
| **RBRC** | **MGI:104629** | **Penk** | **14.93** | **13.01** | **13.60** | **13.19** |
| **RBRC** | **MGI:106484** | **Rufy3** | **13.85** | **13.10** | **13.65** | **13.74** |
| **RBRC** | **MGI:108449** | **Adcyap1r1** | **12.85** | **12.62** | **13.80** | **12.10** |
| **RBRC** | **MGI:109147** | **Oxtr** | **13.83** | **14.76** | **12.09** | **12.09** |
| **RBRC** | **MGI:109331** | **Nxn** | **14.63** | **13.01** | **13.65** | **12.78** |
| **RBRC** | **MGI:109523** | **Trpc6** | **13.62** | **11.90** | **12.55** | **12.01** |
| **RBRC** | **MGI:109524** | **Trpc5** | **14.51** | **13.85** | **14.31** | **13.95** |
| **RBRC** | **MGI:109526** | **Trpc3** | **14.76** | **13.06** | **13.60** | **13.00** |
| **RBRC** | **MGI:1095438** | **Slc2a2** | **13.56** | **13.96** | **14.56** | **13.69** |
| **RBRC** | **MGI:1336167** | **Prkab1** | **16.05** | **13.85** | **15.34** | **13.52** |
| **RBRC** | **MGI:1336993** | **Ap4e1** | **16.31** | **11.96** | **16.25** | **13.98** |
| **RBRC** | **MGI:1342542** | **Ikzf3** | **14.41** | **13.61** | **14.09** | **13.61** |
| **RBRC** | **MGI:1346072** | **Psmf1** | **14.97** | **13.87** | **14.95** | **13.61** |
| **RBRC** | **MGI:1353624** | **Apln** | **14.55** | **13.92** | **15.20** | **12.58** |
| **RBRC** | **MGI:1859162** | **Rnf10** | **15.27** | **13.91** | **14.36** | **14.12** |
| **RBRC** | **MGI:1913955** | **Dnase1l2** | **15.80** | **12.89** | **13.57** | **12.58** |
| **RBRC** | **MGI:1914378** | **Ube2g1** | **16.61** | **13.88** | **15.97** | **13.94** |
| **RBRC** | **MGI:1926245** | **Ube2j1** | **17.29** | **12.35** | **14.42** | **12.46** |
| **RBRC** | **MGI:1929185** | **Izumo1r** | **14.99** | **13.09** | **13.90** | **13.20** |
| **RBRC** | **MGI:1931838** | **Dbn1** | **14.49** | **13.69** | **14.30** | **13.22** |
| **RBRC** | **MGI:2140998** | **Ube3c** | **14.53** | **12.98** | **14.23** | **13.48** |
| **RBRC** | **MGI:2153480** | **Atp6v0a4** | **15.12** | **13.83** | **14.34** | **13.83** |
| **RBRC** | **MGI:2158015** | **Rln3** | **13.71** | **13.18** | **12.99** | **12.73** |
| **RBRC** | **MGI:2445153** | **Clspn** | **14.23** | **13.94** | **14.84** | **13.09** |
| **RBRC** | **MGI:2663979** | **Galp** | **14.43** | **13.91** | **14.24** | **13.95** |
| **RBRC** | **MGI:88297** | **Cck** | **15.44** | **13.83** | **14.36** | **13.40** |
| **RBRC** | **MGI:91842** | **D1Pas1** | **17.12** | **13.54** | **14.72** | **12.67** |
| **RBRC** | **MGI:97931** | **Rln1** | **14.66** | **12.89** | **14.73** | **13.20** |
| **RBRC** | **MGI:98326** | **Sst** | **16.40** | **13.04** | **16.07** | **13.64** |
| **TCP** | **MGI:102556** | **Tbx4** | **14.78** | **13.06** | **17.40** | **11.96** |
| **TCP** | **MGI:102806** | **Acvr2a** | **14.78** | **13.05** | **18.00** | **13.79** |
| **TCP** | **MGI:103075** | **Wee1** | **15.63** | **12.51** | **17.81** | **12.01** |
| **TCP** | **MGI:103180** | **Tcf19** | **15.76** | **13.07** | **17.52** | **12.91** |
| **TCP** | **MGI:103286** | **Atp6v0a1** | **17.46** | **12.55** | **18.34** | **13.32** |
| **TCP** | **MGI:105064** | **Slc9a3** | **15.00** | **12.97** | **18.14** | **13.83** |
| **TCP** | **MGI:106354** | **Vps25** | **14.78** | **11.75** | **17.62** | **10.96** |
| **TCP** | **MGI:106562** | **Dbndd2** | **15.30** | **13.08** | **19.39** | **12.95** |
| **TCP** | **MGI:107416** | **Supt4a** | **15.38** | **11.88** | **16.29** | **12.92** |
| **TCP** | **MGI:107448** | **Lyst** | **16.51** | **13.02** | **17.40** | **12.93** |
| **TCP** | **MGI:107801** | **Atp5b** | **14.78** | **13.02** | **16.57** | **12.94** |
| **TCP** | **MGI:107823** | **Ctsk** | **14.85** | **11.60** | **17.16** | **11.94** |
| **TCP** | **MGI:108027** | **Ptprv** | **16.23** | **13.40** | **17.46** | **12.40** |
| **TCP** | **MGI:108081** | **Sypl** | **16.80** | **11.85** | **19.73** | **13.09** |
| **TCP** | **MGI:109324** | **Fadd** | **14.78** | **11.49** | **18.47** | **11.97** |
| **TCP** | **MGI:109331** | **Nxn** | **14.78** | **11.83** | **17.94** | **11.65** |
| **TCP** | **MGI:109544** | **Aim1** | **15.81** | **12.83** | **16.31** | **13.93** |
| **TCP** | **MGI:109573** | **Tep1** | **19.59** | **13.26** | **15.51** | **13.84** |
| **TCP** | **MGI:109618** | **Atp6v1b2** | **16.59** | **13.06** | **17.54** | **12.93** |
| **TCP** | **MGI:1098274** | **Cyp27b1** | **14.78** | **11.71** | **16.33** | **11.83** |
| **TCP** | **MGI:1098748** | **Ctdsp2** | **16.09** | **13.67** | **19.46** | **12.89** |
| **TCP** | **MGI:1100514** | **Pnn** | **14.78** | **12.31** | **19.21** | **12.89** |
| **TCP** | **MGI:1194508** | **Ddost** | **18.26** | **12.54** | **17.76** | **12.51** |
| **TCP** | **MGI:1195262** | **Man2b2** | **14.78** | **12.94** | **16.67** | **12.96** |
| **TCP** | **MGI:1195985** | **Cbx4** | **13.38** | **11.34** | **16.51** | **11.35** |
| **TCP** | **MGI:1197006** | **Pipox** | **15.83** | **11.67** | **17.86** | **13.60** |
| **TCP** | **MGI:1203729** | **Pik3c2a** | **14.78** | **11.74** | **14.90** | **11.99** |
| **TCP** | **MGI:1289288** | **Slc52a2** | **14.78** | **13.00** | **17.76** | **12.96** |
| **TCP** | **MGI:1309469** | **Casq2** | **16.29** | **13.03** | **16.87** | **12.94** |
| **TCP** | **MGI:1309478** | **Baz1a** | **17.40** | **13.04** | **17.68** | **13.99** |
| **TCP** | **MGI:1316714** | **Cox7a1** | **15.76** | **13.04** | **17.82** | **11.89** |
| **TCP** | **MGI:1330838** | **Lgmn** | **15.86** | **11.84** | **17.36** | **13.17** |
| **TCP** | **MGI:1337104** | **Eya4** | **13.50** | **11.31** | **17.72** | **11.04** |
| **TCP** | **MGI:1338944** | **Acvr1b** | **15.01** | **13.76** | **16.93** | **12.92** |
| **TCP** | **MGI:1342276** | **Mfap4** | **14.70** | **11.86** | **16.67** | **12.02** |
| **TCP** | **MGI:1343085** | **Spop** | **16.93** | **13.94** | **20.21** | **13.71** |
| **TCP** | **MGI:1344414** | **Sra1** | **14.78** | **11.73** | **18.70** | **13.88** |
| **TCP** | **MGI:1346349** | **Sh3bp2** | **16.37** | **13.07** | **17.73** | **12.96** |
| **TCP** | **MGI:1347054** | **Gnpda1** | **14.79** | **11.80** | **18.82** | **12.89** |
| **TCP** | **MGI:1347071** | **Zfp260** | **15.07** | **12.67** | **17.88** | **13.00** |
| **TCP** | **MGI:1353633** | **Fus** | **14.78** | **12.36** | **16.80** | **11.82** |
| **TCP** | **MGI:1858494** | **Bok** | **14.76** | **11.69** | **18.71** | **13.74** |
| **TCP** | **MGI:1858952** | **Arhgef5** | **14.78** | **11.85** | **16.18** | **12.82** |
| **TCP** | **MGI:1859162** | **Rnf10** | **13.34** | **11.52** | **18.36** | **11.17** |
| **TCP** | **MGI:1860138** | **Gtpbp2** | **17.38** | **13.00** | **19.16** | **13.64** |
| **TCP** | **MGI:1861729** | **Abcb9** | **16.71** | **11.61** | **17.39** | **11.95** |
| **TCP** | **MGI:1891341** | **Lpin2** | **15.89** | **11.91** | **16.48** | **12.03** |
| **TCP** | **MGI:1913416** | **S100a14** | **15.54** | **12.37** | **19.14** | **12.88** |
| **TCP** | **MGI:1913711** | **Ptpmt1** | **14.70** | **11.77** | **17.75** | **10.85** |
| **TCP** | **MGI:1913955** | **Dnase1l2** | **14.78** | **11.36** | **17.55** | **11.07** |
| **TCP** | **MGI:1914000** | **Fam210a** | **16.59** | **11.85** | **15.45** | **10.92** |
| **TCP** | **MGI:1914040** | **Grtp1** | **16.27** | **12.96** | **17.43** | **12.92** |
| **TCP** | **MGI:1915138** | **Tmem100** | **14.78** | **11.31** | **18.33** | **12.54** |
| **TCP** | **MGI:1915213** | **Npc2** | **14.78** | **11.51** | **17.64** | **12.50** |
| **TCP** | **MGI:1915283** | **Cox19** | **14.78** | **12.23** | **18.89** | **11.91** |
| **TCP** | **MGI:1915671** | **Lmbrd1** | **14.78** | **11.56** | **15.89** | **11.34** |
| **TCP** | **MGI:1916419** | **Fcmr** | **15.96** | **11.89** | **16.68** | **11.77** |
| **TCP** | **MGI:1917167** | **Nabp2** | **16.64** | **13.03** | **19.39** | **12.93** |
| **TCP** | **MGI:1918788** | **Fbxo9** | **14.72** | **11.86** | **16.80** | **12.88** |
| **TCP** | **MGI:1919201** | **Gpc2** | **14.78** | **11.74** | **17.01** | **11.96** |
| **TCP** | **MGI:1919684** | **Lypd3** | **14.78** | **11.89** | **16.65** | **11.34** |
| **TCP** | **MGI:1919918** | **Skida1** | **15.69** | **13.03** | **18.00** | **12.94** |
| **TCP** | **MGI:1920180** | **Ppp2r2b** | **14.78** | **11.90** | **17.24** | **11.65** |
| **TCP** | **MGI:1920185** | **Ddx41** | **14.78** | **11.78** | **16.14** | **11.14** |
| **TCP** | **MGI:1921084** | **Atp6v1d** | **14.62** | **12.87** | **16.88** | **12.95** |
| **TCP** | **MGI:1921293** | **Pex26** | **14.78** | **13.05** | **18.47** | **12.84** |
| **TCP** | **MGI:1921499** | **Lrrc2** | **14.78** | **11.82** | **17.16** | **11.85** |
| **TCP** | **MGI:1922730** | **1700003F12Rik** | **14.78** | **11.31** | **17.97** | **11.00** |
| **TCP** | **MGI:1922814** | **Rsph9** | **14.78** | **11.70** | **17.75** | **11.22** |
| **TCP** | **MGI:1923428** | **Coa5** | **14.78** | **11.66** | **16.63** | **11.43** |
| **TCP** | **MGI:1923434** | **Abca6** | **15.70** | **12.93** | **17.80** | **13.92** |
| **TCP** | **MGI:1923520** | **Fam49b** | **14.66** | **11.31** | **18.41** | **12.90** |
| **TCP** | **MGI:1925616** | **Gng13** | **14.78** | **11.31** | **19.19** | **12.95** |
| **TCP** | **MGI:1925927** | **Rnf20** | **16.92** | **13.02** | **19.11** | **13.88** |
| **TCP** | **MGI:1926142** | **Crispld2** | **15.86** | **12.71** | **18.07** | **11.97** |
| **TCP** | **MGI:1927073** | **Slc29a1** | **18.03** | **13.42** | **16.12** | **11.67** |
| **TCP** | **MGI:1930088** | **Clcf1** | **14.78** | **13.06** | **19.13** | **11.77** |
| **TCP** | **MGI:1931838** | **Dbn1** | **14.78** | **11.77** | **17.70** | **11.95** |
| **TCP** | **MGI:2137586** | **Dtnbp1** | **16.56** | **13.08** | **17.88** | **13.89** |
| **TCP** | **MGI:2139667** | **Dpyd** | **16.26** | **11.74** | **18.18** | **13.12** |
| **TCP** | **MGI:2142527** | **Exoc8** | **15.80** | **12.21** | **20.13** | **12.35** |
| **TCP** | **MGI:2145369** | **Epdr1** | **15.38** | **11.59** | **15.50** | **11.97** |
| **TCP** | **MGI:2145950** | **Scrib** | **16.35** | **11.80** | **17.33** | **13.80** |
| **TCP** | **MGI:2147627** | **Yars** | **14.91** | **11.78** | **16.95** | **12.76** |
| **TCP** | **MGI:2154658** | **Ubox5** | **16.77** | **11.87** | **18.73** | **12.90** |
| **TCP** | **MGI:2159324** | **Cln6** | **17.43** | **13.27** | **17.84** | **13.85** |
| **TCP** | **MGI:2182928** | **Adgrf5** | **14.78** | **11.85** | **15.77** | **11.88** |
| **TCP** | **MGI:2384801** | **Gps1** | **14.78** | **11.86** | **18.86** | **11.96** |
| **TCP** | **MGI:2384891** | **Rhbdl1** | **17.97** | **13.94** | **19.41** | **11.28** |
| **TCP** | **MGI:2385191** | **Gtf2b** | **16.34** | **13.03** | **19.29** | **14.34** |
| **TCP** | **MGI:2385276** | **Kctd15** | **16.74** | **12.95** | **17.99** | **12.92** |
| **TCP** | **MGI:2387356** | **Ggnbp2** | **14.78** | **12.05** | **19.06** | **12.28** |
| **TCP** | **MGI:2443016** | **Ifi44** | **15.68** | **11.68** | **19.85** | **12.91** |
| **TCP** | **MGI:2443207** | **Plekhm1** | **14.78** | **11.83** | **16.87** | **11.89** |
| **TCP** | **MGI:2448530** | **Vps13d** | **16.84** | **11.84** | **17.57** | **12.02** |
| **TCP** | **MGI:2448567** | **Chd2** | **17.79** | **12.26** | **18.29** | **12.03** |
| **TCP** | **MGI:2449119** | **A2m** | **14.95** | **11.81** | **16.30** | **12.82** |
| **TCP** | **MGI:2449939** | **Zgpat** | **14.78** | **11.90** | **17.21** | **13.08** |
| **TCP** | **MGI:2667185** | **Myo18a** | **18.78** | **12.62** | **18.60** | **14.00** |
| **TCP** | **MGI:2669829** | **D630045J12Rik** | **15.64** | **11.76** | **19.74** | **13.87** |
| **TCP** | **MGI:2679336** | **Satb2** | **16.95** | **13.05** | **19.28** | **13.21** |
| **TCP** | **MGI:2685530** | **Gm684** | **17.24** | **12.20** | **14.52** | **13.37** |
| **TCP** | **MGI:3039593** | **Tox3** | **15.88** | **12.43** | **18.77** | **13.04** |
| **TCP** | **MGI:3039628** | **Rsad1** | **16.61** | **12.94** | **18.63** | **12.95** |
| **TCP** | **MGI:3693832** | **Aktip** | **13.04** | **11.83** | **16.50** | **11.43** |
| **TCP** | **MGI:700009** | **Sh3gl2** | **14.78** | **13.02** | **17.03** | **12.54** |
| **TCP** | **MGI:87968** | **Ahcy** | **14.78** | **12.99** | **19.16** | **12.64** |
| **TCP** | **MGI:88015** | **Ampd1** | **14.78** | **13.27** | **17.20** | **12.93** |
| **TCP** | **MGI:88357** | **Cdk4** | **15.53** | **11.86** | **18.58** | **12.98** |
| **TCP** | **MGI:88393** | **Chd1** | **15.52** | **11.73** | **17.57** | **11.42** |
| **TCP** | **MGI:88473** | **Cox4i1** | **14.80** | **11.82** | **17.45** | **12.93** |
| **TCP** | **MGI:88555** | **Ctla2b** | **16.16** | **12.19** | **16.96** | **10.90** |
| **TCP** | **MGI:94869** | **Dcc** | **14.67** | **11.31** | **19.75** | **12.89** |
| **TCP** | **MGI:94890** | **Dhfr** | **16.10** | **12.62** | **18.27** | **12.89** |
| **TCP** | **MGI:95295** | **Egr1** | **14.78** | **11.87** | **17.17** | **11.98** |
| **TCP** | **MGI:95414** | **Ercc3** | **16.06** | **11.78** | **18.73** | **13.96** |
| **TCP** | **MGI:95522** | **Fgfr1** | **14.89** | **12.72** | **17.76** | **11.92** |
| **TCP** | **MGI:95636** | **Galc** | **15.37** | **11.85** | **16.42** | **13.90** |
| **TCP** | **MGI:95654** | **Gart** | **14.78** | **11.83** | **16.26** | **11.96** |
| **TCP** | **MGI:96413** | **Idh1** | **21.55** | **13.02** | **19.42** | **13.93** |
| **TCP** | **MGI:97621** | **Plk1** | **17.27** | **11.84** | **15.86** | **11.41** |
| **TCP** | **MGI:97788** | **Psph** | **14.78** | **11.77** | **17.51** | **11.29** |
| **TCP** | **MGI:97838** | **Eprs** | **16.41** | **13.32** | **19.80** | **13.90** |
| **TCP** | **MGI:98960** | **Wnt6** | **14.78** | **12.40** | **16.14** | **12.91** |
| **TCP** | **MGI:99907** | **Plod1** | **15.69** | **13.03** | **18.17** | **13.79** |
| **TCP** | **MGI:99960** | **Ewsr1** | **16.10** | **11.83** | **19.99** | **13.91** |
| **HMGU** | **MGI:101932** | **Cpe** | **13.20** | **12.04** | **14.98** | **12.81** |
| **HMGU** | **MGI:102772** | **Gbp2** | **13.27** | **12.33** | **14.16** | **12.10** |
| **HMGU** | **MGI:102805** | **Entpd1** | **12.33** | **12.29** | **14.58** | **12.09** |
| **HMGU** | **MGI:102849** | **Klkb1** | **12.60** | **12.20** | **12.59** | **11.01** |
| **HMGU** | **MGI:103224** | **Fdx1** | **13.34** | **11.93** | **14.87** | **12.02** |
| **HMGU** | **MGI:103225** | **Cyp4b1** | **12.72** | **12.22** | **16.67** | **11.99** |
| **HMGU** | **MGI:103296** | **Nfatc3** | **13.63** | **12.51** | **14.26** | **11.99** |
| **HMGU** | **MGI:103300** | **Rbl1** | **15.42** | **11.69** | **15.50** | **12.05** |
| **HMGU** | **MGI:104559** | **Rcn1** | **12.88** | **12.34** | **15.13** | **12.90** |
| **HMGU** | **MGI:104576** | **Lct** | **13.96** | **11.47** | **15.77** | **12.04** |
| **HMGU** | **MGI:104630** | **Wars** | **13.60** | **11.25** | **16.34** | **11.99** |
| **HMGU** | **MGI:104661** | **Rora** | **12.39** | **12.27** | **14.05** | **12.26** |
| **HMGU** | **MGI:104672** | **Tfap2b** | **14.16** | **11.48** | **16.62** | **12.70** |
| **HMGU** | **MGI:104897** | **Rest** | **13.08** | **12.51** | **17.36** | **11.59** |
| **HMGU** | **MGI:106091** | **Cyp7a1** | **13.64** | **11.71** | **16.88** | **12.44** |
| **HMGU** | **MGI:106100** | **Etfdh** | **14.20** | **11.35** | **14.54** | **12.03** |
| **HMGU** | **MGI:106181** | **Mybbp1a** | **12.72** | **11.79** | **14.49** | **11.22** |
| **HMGU** | **MGI:106645** | **Enpep** | **14.62** | **12.20** | **15.34** | **12.46** |
| **HMGU** | **MGI:107379** | **Gstt1** | **15.45** | **11.84** | **16.60** | **12.78** |
| **HMGU** | **MGI:107796** | **Akr7a5** | **14.25** | **11.83** | **15.05** | **12.13** |
| **HMGU** | **MGI:109211** | **Pkn2** | **14.45** | **12.17** | **16.14** | **12.97** |
| **HMGU** | **MGI:109331** | **Nxn** | **13.01** | **11.84** | **14.26** | **11.83** |
| **HMGU** | **MGI:109431** | **Raet1c** | **14.24** | **11.83** | **15.53** | **12.65** |
| **HMGU** | **MGI:109555** | **Psmc2** | **14.13** | **11.41** | **16.49** | **12.86** |
| **HMGU** | **MGI:1096317** | **Eef1a2** | **14.69** | **11.54** | **16.25** | **12.87** |
| **HMGU** | **MGI:1096574** | **Car4** | **14.27** | **12.47** | **15.83** | **12.17** |
| **HMGU** | **MGI:1098230** | **Cenpe** | **12.73** | **12.04** | **14.59** | **12.00** |
| **HMGU** | **MGI:1098597** | **Ift81** | **13.06** | **11.75** | **15.85** | **12.07** |
| **HMGU** | **MGI:1196419** | **Ccpg1** | **14.00** | **11.58** | **14.92** | **12.25** |
| **HMGU** | **MGI:1201378** | **Anxa3** | **13.33** | **11.96** | **14.83** | **12.06** |
| **HMGU** | **MGI:1201682** | **Kif20a** | **13.06** | **12.15** | **15.00** | **11.99** |
| **HMGU** | **MGI:1202298** | **Nmt2** | **13.73** | **11.92** | **16.39** | **11.96** |
| **HMGU** | **MGI:1270128** | **Usp12** | **12.91** | **12.38** | **14.81** | **12.01** |
| **HMGU** | **MGI:1277180** | **Ifi27** | **13.94** | **12.03** | **15.70** | **12.04** |
| **HMGU** | **MGI:1278321** | **Epc2** | **12.75** | **11.77** | **14.89** | **12.09** |
| **HMGU** | **MGI:1309467** | **Gstm6** | **13.44** | **12.12** | **15.00** | **12.02** |
| **HMGU** | **MGI:1309515** | **Gsta4** | **12.76** | **11.59** | **14.74** | **11.95** |
| **HMGU** | **MGI:1309998** | **Marco** | **15.31** | **11.38** | **15.06** | **12.01** |
| **HMGU** | **MGI:1313312** | **Khdrbs3** | **13.03** | **11.74** | **16.40** | **11.94** |
| **HMGU** | **MGI:1314882** | **Hipk3** | **13.98** | **11.39** | **15.48** | **11.97** |
| **HMGU** | **MGI:1329021** | **Msh5** | **14.42** | **12.34** | **16.61** | **12.39** |
| **HMGU** | **MGI:1333865** | **Rfxank** | **14.11** | **11.36** | **14.71** | **12.03** |
| **HMGU** | **MGI:1336155** | **Lss** | **13.66** | **12.11** | **15.26** | **12.04** |
| **HMGU** | **MGI:1336167** | **Prkab1** | **13.69** | **11.62** | **15.31** | **11.99** |
| **HMGU** | **MGI:1336894** | **Mocs2** | **14.02** | **12.53** | **15.35** | **12.40** |
| **HMGU** | **MGI:1336993** | **Ap4e1** | **12.73** | **11.47** | **14.81** | **11.98** |
| **HMGU** | **MGI:1338017** | **Bex2** | **13.98** | **11.95** | **15.54** | **12.86** |
| **HMGU** | **MGI:1338883** | **Gfpt2** | **13.13** | **11.90** | **14.91** | **12.02** |
| **HMGU** | **MGI:1339957** | **P2rx7** | **12.59** | **12.43** | **14.82** | **11.80** |
| **HMGU** | **MGI:1339968** | **Cth** | **13.56** | **11.99** | **15.38** | **12.03** |
| **HMGU** | **MGI:1340024** | **Aldh1l1** | **12.72** | **11.45** | **14.56** | **12.17** |
| **HMGU** | **MGI:1341204** | **Aqp6** | **13.52** | **11.46** | **16.29** | **12.06** |
| **HMGU** | **MGI:1341724** | **Ggps1** | **12.65** | **11.26** | **15.12** | **12.01** |
| **HMGU** | **MGI:1342274** | **Slc25a15** | **14.16** | **11.40** | **15.21** | **12.06** |
| **HMGU** | **MGI:1343086** | **Pkig** | **13.46** | **11.85** | **14.50** | **12.03** |
| **HMGU** | **MGI:1343098** | **Aif1** | **13.87** | **11.79** | **15.25** | **12.07** |
| **HMGU** | **MGI:1345189** | **Zfp119a** | **12.64** | **11.89** | **14.90** | **12.06** |
| **HMGU** | **MGI:1346327** | **Fyb** | **12.61** | **12.16** | **14.73** | **12.05** |
| **HMGU** | **MGI:1346879** | **Map3k10** | **12.12** | **12.43** | **14.46** | **12.02** |
| **HMGU** | **MGI:1347059** | **Decr2** | **15.08** | **11.78** | **14.73** | **12.05** |
| **HMGU** | **MGI:1347072** | **Nubp2** | **13.46** | **12.38** | **15.70** | **12.01** |
| **HMGU** | **MGI:1347352** | **Hunk** | **14.09** | **12.00** | **14.62** | **14.06** |
| **HMGU** | **MGI:1347522** | **Pla2g10** | **12.47** | **12.53** | **15.08** | **12.87** |
| **HMGU** | **MGI:1349165** | **Sdc2** | **12.47** | **11.41** | **14.87** | **11.99** |
| **HMGU** | **MGI:1349448** | **Cenph** | **13.09** | **11.77** | **15.11** | **12.02** |
| **HMGU** | **MGI:1351619** | **Abcb11** | **14.39** | **12.49** | **15.07** | **12.93** |
| **HMGU** | **MGI:1351627** | **Pdhx** | **13.61** | **12.05** | **15.84** | **12.04** |
| **HMGU** | **MGI:1351628** | **Rps26** | **13.91** | **11.30** | **14.89** | **11.19** |
| **HMGU** | **MGI:1351825** | **Cstf3** | **13.44** | **12.30** | **14.83** | **12.03** |
| **HMGU** | **MGI:1353511** | **Sspn** | **13.68** | **11.74** | **15.45** | **12.16** |
| **HMGU** | **MGI:1353606** | **Siva1** | **13.01** | **11.63** | **14.89** | **12.03** |
| **HMGU** | **MGI:1858208** | **Ech1** | **14.95** | **11.92** | **16.38** | **12.92** |
| **HMGU** | **MGI:1858910** | **Jmjd6** | **15.01** | **12.36** | **15.29** | **12.10** |
| **HMGU** | **MGI:1858961** | **Prc1** | **13.96** | **12.06** | **16.01** | **12.05** |
| **HMGU** | **MGI:1859162** | **Rnf10** | **13.42** | **11.62** | **15.16** | **11.96** |
| **HMGU** | **MGI:1891369** | **Capn12** | **13.42** | **11.46** | **16.62** | **12.66** |
| **HMGU** | **MGI:1891748** | **Maea** | **14.29** | **11.87** | **15.60** | **12.10** |
| **HMGU** | **MGI:1891827** | **Gde1** | **13.48** | **12.13** | **15.25** | **11.98** |
| **HMGU** | **MGI:1891828** | **Becn1** | **14.20** | **12.01** | **15.64** | **12.93** |
| **HMGU** | **MGI:1913310** | **Cystm1** | **13.42** | **12.37** | **15.77** | **12.93** |
| **HMGU** | **MGI:1913337** | **Emc3** | **13.96** | **12.20** | **15.90** | **12.04** |
| **HMGU** | **MGI:1913368** | **Sarnp** | **13.46** | **11.65** | **15.19** | **12.03** |
| **HMGU** | **MGI:1913415** | **Bccip** | **13.61** | **11.51** | **15.52** | **12.04** |
| **HMGU** | **MGI:1913468** | **Ndufb9** | **14.57** | **11.44** | **15.06** | **12.02** |
| **HMGU** | **MGI:1913536** | **Sec11c** | **13.95** | **11.22** | **14.27** | **11.98** |
| **HMGU** | **MGI:1913538** | **Pdcd5** | **14.15** | **12.13** | **15.10** | **11.50** |
| **HMGU** | **MGI:1913687** | **Fis1** | **14.18** | **11.88** | **15.36** | **12.07** |
| **HMGU** | **MGI:1913838** | **Cmpk1** | **13.96** | **11.40** | **15.10** | **12.02** |
| **HMGU** | **MGI:1913945** | **Aspn** | **13.48** | **12.25** | **15.73** | **12.12** |
| **HMGU** | **MGI:1913955** | **Dnase1l2** | **13.59** | **11.38** | **15.71** | **11.97** |
| **HMGU** | **MGI:1914080** | **Nacc1** | **13.61** | **11.72** | **16.49** | **12.63** |
| **HMGU** | **MGI:1914258** | **Yae1d1** | **13.32** | **11.50** | **16.11** | **12.05** |
| **HMGU** | **MGI:1914342** | **Gatm** | **14.50** | **12.17** | **15.27** | **12.00** |
| **HMGU** | **MGI:1914457** | **Lsm1** | **15.00** | **12.50** | **15.73** | **12.23** |
| **HMGU** | **MGI:1914491** | **Smc6** | **13.99** | **11.60** | **13.62** | **12.02** |
| **HMGU** | **MGI:1914502** | **Cap2** | **12.48** | **12.17** | **14.78** | **12.42** |
| **HMGU** | **MGI:1914523** | **Ndufa10** | **13.83** | **11.48** | **14.52** | **12.01** |
| **HMGU** | **MGI:1914672** | **Dhdds** | **13.22** | **11.61** | **15.34** | **12.03** |
| **HMGU** | **MGI:1914723** | **Slc47a1** | **12.88** | **12.07** | **14.33** | **12.01** |
| **HMGU** | **MGI:1914780** | **Uqcrb** | **12.79** | **11.56** | **14.20** | **11.98** |
| **HMGU** | **MGI:1914917** | **Alkbh8** | **14.28** | **11.69** | **16.22** | **12.02** |
| **HMGU** | **MGI:1914930** | **Sdhb** | **12.80** | **11.95** | **15.73** | **12.15** |
| **HMGU** | **MGI:1915045** | **Rnls** | **14.21** | **12.14** | **15.77** | **12.05** |
| **HMGU** | **MGI:1915148** | **Pef1** | **12.23** | **11.46** | **14.38** | **12.01** |
| **HMGU** | **MGI:1915192** | **Atp5g2** | **12.92** | **11.64** | **14.95** | **12.02** |
| **HMGU** | **MGI:1915301** | **Nutf2** | **13.44** | **11.76** | **15.28** | **12.20** |
| **HMGU** | **MGI:1915625** | **Ndufa8** | **12.22** | **12.01** | **13.91** | **12.09** |
| **HMGU** | **MGI:1915778** | **Smim6** | **13.56** | **12.10** | **14.17** | **12.85** |
| **HMGU** | **MGI:1915918** | **Klk5** | **13.04** | **11.57** | **14.90** | **11.92** |
| **HMGU** | **MGI:1916082** | **Ldah** | **13.20** | **11.55** | **15.99** | **11.90** |
| **HMGU** | **MGI:1916198** | **Fam216a** | **14.52** | **12.08** | **14.80** | **11.48** |
| **HMGU** | **MGI:1916214** | **Ctc1** | **15.60** | **12.60** | **17.57** | **12.93** |
| **HMGU** | **MGI:1916216** | **Ngdn** | **15.36** | **11.65** | **17.51** | **12.96** |
| **HMGU** | **MGI:1916308** | **Oscp1** | **14.91** | **12.03** | **14.54** | **12.03** |
| **HMGU** | **MGI:1916625** | **Btbd9** | **12.73** | **11.63** | **15.60** | **12.83** |
| **HMGU** | **MGI:1916658** | **Dnajc17** | **12.16** | **11.99** | **13.44** | **12.00** |
| **HMGU** | **MGI:1916679** | **Me3** | **13.59** | **12.23** | **15.54** | **12.07** |
| **HMGU** | **MGI:1916784** | **Avpi1** | **12.85** | **12.23** | **14.94** | **12.04** |
| **HMGU** | **MGI:1916812** | **Cdk13** | **14.74** | **11.26** | **16.31** | **12.96** |
| **HMGU** | **MGI:1916983** | **Paox** | **13.65** | **11.54** | **15.22** | **11.96** |
| **HMGU** | **MGI:1917226** | **Galk2** | **13.60** | **11.67** | **15.23** | **12.08** |
| **HMGU** | **MGI:1917379** | **Slc44a4** | **15.34** | **11.52** | **14.76** | **12.02** |
| **HMGU** | **MGI:1917436** | **Fam162a** | **13.30** | **11.94** | **15.12** | **11.99** |
| **HMGU** | **MGI:1917623** | **Gpatch2l** | **13.67** | **11.57** | **14.87** | **12.50** |
| **HMGU** | **MGI:1917680** | **Tbce** | **13.48** | **11.78** | **14.85** | **12.02** |
| **HMGU** | **MGI:1917761** | **Eef2kmt** | **15.35** | **11.78** | **16.99** | **12.61** |
| **HMGU** | **MGI:1918019** | **Nolc1** | **14.16** | **11.76** | **14.32** | **12.75** |
| **HMGU** | **MGI:1918419** | **Nbas** | **14.85** | **12.04** | **15.82** | **12.14** |
| **HMGU** | **MGI:1919008** | **Vwa8** | **13.17** | **12.08** | **15.07** | **11.98** |
| **HMGU** | **MGI:1919103** | **Pdia6** | **13.85** | **12.03** | **15.72** | **12.02** |
| **HMGU** | **MGI:1919292** | **Cotl1** | **13.91** | **12.27** | **14.22** | **12.04** |
| **HMGU** | **MGI:1919301** | **Pard3b** | **13.37** | **11.29** | **15.35** | **12.79** |
| **HMGU** | **MGI:1919387** | **Wdsub1** | **13.12** | **12.00** | **15.12** | **11.98** |
| **HMGU** | **MGI:1919912** | **Dis3** | **13.00** | **11.47** | **13.64** | **10.99** |
| **HMGU** | **MGI:1920014** | **Csnk1g2** | **14.94** | **11.91** | **13.93** | **12.03** |
| **HMGU** | **MGI:1920344** | **Sgip1** | **13.28** | **11.69** | **16.85** | **12.13** |
| **HMGU** | **MGI:1920524** | **Gpbp1** | **13.32** | **11.24** | **15.44** | **12.96** |
| **HMGU** | **MGI:1920740** | **Mipol1** | **12.53** | **12.39** | **14.72** | **11.97** |
| **HMGU** | **MGI:1921392** | **Lonp1** | **13.72** | **12.39** | **15.66** | **11.96** |
| **HMGU** | **MGI:1921627** | **Hsf2bp** | **12.79** | **12.54** | **14.46** | **11.93** |
| **HMGU** | **MGI:1921701** | **Pgs1** | **13.16** | **12.41** | **14.66** | **12.19** |
| **HMGU** | **MGI:1921765** | **Cdkal1** | **12.94** | **12.30** | **13.75** | **12.00** |
| **HMGU** | **MGI:1921840** | **Ankrd10** | **14.42** | **11.74** | **15.67** | **12.96** |
| **HMGU** | **MGI:1922462** | **Rnf121** | **14.57** | **12.25** | **14.24** | **12.04** |
| **HMGU** | **MGI:1922546** | **Fgfr1op** | **13.36** | **12.09** | **15.10** | **12.04** |
| **HMGU** | **MGI:1922725** | **Oplah** | **13.36** | **11.67** | **15.94** | **12.01** |
| **HMGU** | **MGI:1922945** | **Rilpl1** | **12.80** | **11.53** | **14.95** | **12.00** |
| **HMGU** | **MGI:1923275** | **Cant1** | **13.45** | **11.82** | **15.61** | **11.59** |
| **HMGU** | **MGI:1923363** | **Lpo** | **12.09** | **12.07** | **14.52** | **11.99** |
| **HMGU** | **MGI:1923523** | **Ndfip2** | **13.33** | **12.39** | **14.74** | **12.01** |
| **HMGU** | **MGI:1923531** | **Tax1bp3** | **15.10** | **11.34** | **16.35** | **12.48** |
| **HMGU** | **MGI:1923616** | **Mtif3** | **14.96** | **12.03** | **16.58** | **12.06** |
| **HMGU** | **MGI:1923649** | **Il31** | **15.39** | **12.09** | **15.19** | **12.10** |
| **HMGU** | **MGI:1923714** | **Casc5** | **14.19** | **11.39** | **13.25** | **12.06** |
| **HMGU** | **MGI:1923757** | **Aoc1** | **12.71** | **11.63** | **15.28** | **12.00** |
| **HMGU** | **MGI:1923998** | **Pbrm1** | **14.43** | **11.73** | **16.03** | **12.20** |
| **HMGU** | **MGI:1924182** | **Arfip2** | **13.25** | **11.46** | **14.83** | **11.02** |
| **HMGU** | **MGI:1924567** | **Fam73a** | **12.70** | **11.36** | **14.50** | **11.96** |
| **HMGU** | **MGI:1924712** | **Tmem116** | **13.66** | **11.30** | **14.50** | **11.97** |
| **HMGU** | **MGI:1925188** | **Fam53b** | **12.22** | **12.32** | **13.87** | **12.02** |
| **HMGU** | **MGI:1927594** | **Itm2c** | **12.83** | **11.67** | **13.96** | **12.01** |
| **HMGU** | **MGI:1927669** | **Cyp4f14** | **13.73** | **11.78** | **15.03** | **12.05** |
| **HMGU** | **MGI:1928369** | **Slc2a5** | **14.49** | **12.09** | **15.74** | **12.04** |
| **HMGU** | **MGI:1928483** | **Stx5a** | **14.14** | **11.97** | **17.19** | **12.95** |
| **HMGU** | **MGI:1931825** | **Chst5** | **13.72** | **12.20** | **15.02** | **12.21** |
| **HMGU** | **MGI:1931838** | **Dbn1** | **13.22** | **11.98** | **14.52** | **12.42** |
| **HMGU** | **MGI:1933126** | **Cdk5rap3** | **13.67** | **12.14** | **14.78** | **11.99** |
| **HMGU** | **MGI:1933395** | **Lactb** | **13.69** | **12.35** | **15.50** | **11.89** |
| **HMGU** | **MGI:2137026** | **P2rx5** | **13.11** | **12.11** | **14.62** | **12.29** |
| **HMGU** | **MGI:2138811** | **Cdc123** | **13.24** | **11.52** | **15.14** | **12.01** |
| **HMGU** | **MGI:2139806** | **Spg20** | **12.04** | **12.51** | **14.62** | **11.99** |
| **HMGU** | **MGI:2140260** | **Pcsk9** | **14.83** | **11.91** | **16.00** | **12.09** |
| **HMGU** | **MGI:2144041** | **Wsb2** | **15.57** | **11.65** | **14.36** | **11.66** |
| **HMGU** | **MGI:2144474** | **Smek2** | **14.62** | **12.44** | **15.26** | **12.57** |
| **HMGU** | **MGI:2144837** | **Ptpn23** | **13.10** | **11.97** | **13.16** | **12.00** |
| **HMGU** | **MGI:2146553** | **Osbpl11** | **14.07** | **11.87** | **15.33** | **11.98** |
| **HMGU** | **MGI:2148931** | **Ly6g6d** | **14.13** | **11.94** | **14.41** | **11.99** |
| **HMGU** | **MGI:2152453** | **Gsk3a** | **15.51** | **11.96** | **14.69** | **12.52** |
| **HMGU** | **MGI:2152889** | **Dner** | **12.85** | **11.73** | **15.34** | **12.02** |
| **HMGU** | **MGI:2153463** | **Rxfp2** | **14.14** | **11.82** | **15.98** | **12.03** |
| **HMGU** | **MGI:2179733** | **Mpst** | **13.88** | **11.21** | **14.48** | **12.00** |
| **HMGU** | **MGI:2181074** | **Acbd3** | **12.77** | **11.76** | **15.36** | **12.00** |
| **HMGU** | **MGI:2181202** | **Pfkfb3** | **14.03** | **12.16** | **13.98** | **12.19** |
| **HMGU** | **MGI:2384034** | **Alkbh1** | **13.80** | **11.47** | **14.44** | **11.99** |
| **HMGU** | **MGI:2384860** | **Slc44a3** | **14.68** | **11.91** | **15.19** | **12.67** |
| **HMGU** | **MGI:2386323** | **Acmsd** | **14.25** | **11.79** | **15.41** | **11.89** |
| **HMGU** | **MGI:2442230** | **Mfn2** | **14.03** | **11.31** | **15.58** | **12.07** |
| **HMGU** | **MGI:2442264** | **Idi1** | **14.09** | **11.61** | **15.01** | **12.04** |
| **HMGU** | **MGI:2442327** | **Xkr5** | **13.52** | **11.95** | **15.16** | **12.01** |
| **HMGU** | **MGI:2442557** | **Frmd5** | **12.19** | **11.92** | **16.01** | **12.42** |
| **HMGU** | **MGI:2443036** | **Dnajc27** | **14.00** | **11.92** | **16.00** | **12.80** |
| **HMGU** | **MGI:2443241** | **Ndufs1** | **13.50** | **11.70** | **14.41** | **11.49** |
| **HMGU** | **MGI:2443671** | **Dlec1** | **12.47** | **12.37** | **15.25** | **12.03** |
| **HMGU** | **MGI:2443967** | **Dpp9** | **12.58** | **12.15** | **14.17** | **12.03** |
| **HMGU** | **MGI:2444207** | **Vps13c** | **13.03** | **11.95** | **15.44** | **12.02** |
| **HMGU** | **MGI:2444631** | **4932438A13Rik** | **14.48** | **12.20** | **15.10** | **11.93** |
| **HMGU** | **MGI:2445289** | **Plac8** | **13.29** | **12.11** | **15.37** | **12.38** |
| **HMGU** | **MGI:2446132** | **Bms1** | **13.09** | **12.09** | **15.95** | **11.98** |
| **HMGU** | **MGI:2446173** | **Farp1** | **13.33** | **11.47** | **15.05** | **12.68** |
| **HMGU** | **MGI:2446175** | **Spryd3** | **13.09** | **12.32** | **14.10** | **11.94** |
| **HMGU** | **MGI:2450151** | **Cog3** | **13.01** | **11.92** | **13.46** | **11.97** |
| **HMGU** | **MGI:2685412** | **Bcl2l15** | **13.52** | **12.15** | **14.32** | **12.53** |
| **HMGU** | **MGI:2686925** | **Cyb561a3** | **13.48** | **11.46** | **15.61** | **12.07** |
| **HMGU** | **MGI:3037150** | **Slc13a5** | **14.59** | **12.20** | **15.12** | **12.02** |
| **HMGU** | **MGI:3039618** | **Zbtb24** | **13.17** | **11.50** | **13.47** | **12.08** |
| **HMGU** | **MGI:3505689** | **Cdsn** | **13.97** | **12.00** | **16.19** | **12.98** |
| **HMGU** | **MGI:3617840** | **Cpa2** | **12.11** | **12.15** | **13.53** | **11.93** |
| **HMGU** | **MGI:3780550** | **Mthfsl** | **12.72** | **11.88** | **14.19** | **12.01** |
| **HMGU** | **MGI:700012** | **Ostf1** | **15.05** | **11.37** | **16.14** | **12.02** |
| **HMGU** | **MGI:87878** | **Apoc4** | **13.50** | **12.03** | **15.55** | **12.04** |
| **HMGU** | **MGI:87914** | **Aspa** | **15.38** | **12.11** | **16.43** | **12.59** |
| **HMGU** | **MGI:88222** | **Tspo** | **12.87** | **11.92** | **14.02** | **12.30** |
| **HMGU** | **MGI:88529** | **Cs** | **13.04** | **12.02** | **14.94** | **12.89** |
| **HMGU** | **MGI:95491** | **Fbp2** | **12.87** | **11.38** | **15.11** | **11.98** |
| **HMGU** | **MGI:95634** | **Gad2** | **13.54** | **12.41** | **17.09** | **13.03** |
| **HMGU** | **MGI:96174** | **Hoxa2** | **13.66** | **11.78** | **14.33** | **11.99** |
| **HMGU** | **MGI:96765** | **Ldlr** | **13.53** | **11.64** | **16.69** | **12.56** |
| **HMGU** | **MGI:96877** | **Klrb1** | **13.57** | **12.03** | **15.42** | **12.17** |
| **HMGU** | **MGI:96973** | **Mgat1** | **13.43** | **12.36** | **14.94** | **12.07** |
| **HMGU** | **MGI:97272** | **Myl2** | **15.15** | **11.45** | **15.25** | **12.04** |
| **HMGU** | **MGI:97489** | **Pax5** | **13.81** | **12.02** | **13.07** | **11.99** |
| **HMGU** | **MGI:97520** | **Pcx** | **13.46** | **11.82** | **14.45** | **11.47** |
| **HMGU** | **MGI:97753** | **Ppy** | **13.69** | **12.38** | **15.52** | **12.01** |
| **HMGU** | **MGI:98038** | **Rpl32** | **14.97** | **12.22** | **16.34** | **13.01** |
| **HMGU** | **MGI:98301** | **Pmel** | **13.68** | **11.30** | **14.67** | **12.04** |
| **HMGU** | **MGI:98483** | **Tap1** | **15.44** | **11.91** | **17.12** | **12.29** |
| **HMGU** | **MGI:98894** | **Ucp1** | **13.48** | **11.76** | **14.09** | **12.15** |
| **HMGU** | **MGI:99432** | **Arf3** | **13.98** | **12.08** | **15.28** | **12.00** |
| **HMGU** | **MGI:99515** | **Syk** | **14.11** | **11.60** | **13.31** | **11.98** |
| **HMGU** | **MGI:99600** | **Aldh2** | **13.57** | **11.88** | **14.54** | **12.49** |
| **HMGU** | **MGI:99663** | **Zfp61** | **15.09** | **11.92** | **15.87** | **12.03** |
| **HMGU** | **MGI:99845** | **Gdi2** | **14.93** | **12.00** | **15.37** | **12.00** |
| **HMGU** | **MGI:99892** | **Lama1** | **13.39** | **11.89** | **14.24** | **12.05** |
