# Supplementary material for: High-throughput discovery of genetic determinants of circadian misalignment
Source: PLoS Genet. 2020 Jan 13;16(1):e1008577. doi: 10.1371/journal.pgen.1008577 (PMC6980734; doi:10.1371/journal.pgen.1008577)
Supplement: S9 Table — (DOCX) [file pgen.1008577.s013.docx]

**S9 Table. Mutant lines existing in at least two centers**

| **gene ID** | **gene Symbol** | **Center** | **Activity** | | | | | **Food intake** | | | |
| --- | --- | --- | --- | --- | --- | --- | --- | --- | --- | --- | --- |
|  |  |  | **Phase** | | **Onset** | | **Phase** | | | **Onset** | |
|  |  |  | **Mutant** | **WT** | **Mutant** | **WT** | **Mutant** | | **WT** | **Mutant** | **WT** |
| **MGI:106441** | **Zranb1** | **WTSI** | **15.60** | **14.67** | **12.52** | **12.67** | **14.77** | | **15.06** | **12.02** | **12.67** |
| **MGI:106441** | **Zranb1** | **ICS** | **12.62** | **13.85** | **11.03** | **11.67** | **14.59** | | **15.34** | **11.08** | **12.71** |
| **MGI:109331** | **Nxn** | **WTSI** | **14.54** | **14.67** | **12.62** | **12.67** | **17.17** | | **15.06** | **12.85** | **12.67** |
| **MGI:109331** | **Nxn** | **ICS** | **14.83** | **13.85** | **11.92** | **11.67** | **13.97** | | **15.34** | **12.12** | **12.71** |
| **MGI:109331** | **Nxn** | **TCP** | **14.78** | **14.99** | **11.83** | **12.65** | **17.94** | | **16.79** | **11.65** | **13.08** |
| **MGI:109331** | **Nxn** | **RBRC** | **14.63** | **15.04** | **13.01** | **13.67** | **13.65** | | **15.01** | **12.78** | **12.99** |
| **MGI:109331** | **Nxn** | **HMGU** | **13.01** | **13.59** | **11.84** | **12.11** | **14.26** | | **15.19** | **11.83** | **13.02** |
| **MGI:1195262** | **Man2b2** | **WTSI** | **15.14** | **14.67** | **12.66** | **12.67** | **17.53** | | **15.06** | **12.99** | **12.67** |
| **MGI:1195262** | **Man2b2** | **TCP** | **14.78** | **14.99** | **12.94** | **12.65** | **16.67** | | **16.79** | **12.96** | **13.08** |
| **MGI:1336993** | **Ap4e1** | **WTSI** | **14.54** | **14.67** | **12.49** | **12.67** | **16.28** | | **15.06** | **12.00** | **12.67** |
| **MGI:1336993** | **Ap4e1** | **ICS** | **12.84** | **13.85** | **12.00** | **11.67** | **16.81** | | **15.34** | **11.99** | **12.71** |
| **MGI:1336993** | **Ap4e1** | **RBRC** | **16.31** | **15.04** | **11.96** | **13.67** | **16.25** | | **15.01** | **13.98** | **12.99** |
| **MGI:1336993** | **Ap4e1** | **HMGU** | **12.73** | **13.59** | **11.47** | **12.11** | **14.81** | | **15.19** | **11.98** | **13.02** |
| **MGI:1859162** | **Rnf10** | **ICS** | **13.11** | **13.85** | **11.98** | **11.67** | **17.79** | | **15.34** | **11.74** | **12.71** |
| **MGI:1859162** | **Rnf10** | **TCP** | **13.34** | **14.99** | **11.52** | **12.65** | **18.36** | | **16.79** | **11.17** | **13.08** |
| **MGI:1859162** | **Rnf10** | **RBRC** | **15.27** | **15.04** | **13.91** | **13.67** | **14.36** | | **15.01** | **14.12** | **12.99** |
| **MGI:1859162** | **Rnf10** | **HMGU** | **13.42** | **13.59** | **11.62** | **12.11** | **15.16** | | **15.19** | **11.96** | **13.02** |
| **MGI:1913955** | **Dnase1l2** | **WTSI** | **14.20** | **14.67** | **12.53** | **12.67** | **16.35** | | **15.06** | **12.99** | **12.67** |
| **MGI:1913955** | **Dnase1l2** | **ICS** | **14.85** | **13.85** | **11.88** | **11.67** | **15.93** | | **15.34** | **11.56** | **12.71** |
| **MGI:1913955** | **Dnase1l2** | **TCP** | **14.78** | **14.99** | **11.36** | **12.65** | **17.55** | | **16.79** | **11.07** | **13.08** |
| **MGI:1913955** | **Dnase1l2** | **RBRC** | **15.80** | **15.04** | **12.89** | **13.67** | **13.57** | | **15.01** | **12.58** | **12.99** |
| **MGI:1913955** | **Dnase1l2** | **HMGU** | **13.59** | **13.59** | **11.38** | **12.11** | **15.71** | | **15.19** | **11.97** | **13.02** |
| **MGI:1919918** | **Skida1** | **WTSI** | **13.88** | **14.67** | **12.53** | **12.67** | **13.32** | | **15.06** | **12.02** | **12.67** |
| **MGI:1919918** | **Skida1** | **TCP** | **15.69** | **14.99** | **13.03** | **12.65** | **18.00** | | **16.79** | **12.94** | **13.08** |
| **MGI:1921084** | **Atp6v1d** | **ICS** | **14.23** | **13.85** | **11.77** | **11.67** | **13.45** | | **15.34** | **11.67** | **12.71** |
| **MGI:1921084** | **Atp6v1d** | **TCP** | **14.62** | **14.99** | **12.87** | **12.65** | **16.88** | | **16.79** | **12.95** | **13.08** |
| **MGI:1931838** | **Dbn1** | **ICS** | **14.04** | **13.85** | **11.05** | **11.67** | **14.89** | | **15.34** | **11.97** | **12.71** |
| **MGI:1931838** | **Dbn1** | **RBRC** | **14.49** | **15.04** | **13.69** | **13.67** | **14.30** | | **15.01** | **13.22** | **12.99** |
| **MGI:1931838** | **Dbn1** | **TCP** | **14.78** | **14.99** | **11.77** | **12.65** | **17.70** | | **16.79** | **11.95** | **13.08** |
| **MGI:1931838** | **Dbn1** | **HMGU** | **13.22** | **13.59** | **11.98** | **12.11** | **14.52** | | **15.19** | **14.42** | **13.02** |
| **MGI:3039628** | **Rsad1** | **WTSI** | **14.54** | **14.67** | **12.62** | **12.67** | **15.70** | | **15.06** | **13.54** | **12.67** |
| **MGI:3039628** | **Rsad1** | **TCP** | **16.61** | **14.99** | **12.94** | **12.65** | **18.63** | | **16.79** | **12.95** | **13.08** |
| **MGI:1336167** | **Prkab1** | **RBRC** | **16.05** | **15.04** | **13.85** | **13.67** | **15.34** | | **15.01** | **13.52** | **12.99** |
| **MGI:1336167** | **Prkab1** | **HMGU** | **13.69** | **13.59** | **11.62** | **12.11** | **15.31** | | **15.19** | **11.99** | **13.02** |
| **MGI:1921765** | **Cdkal1** | **WTSI** | **13.83** | **14.67** | **12.49** | **12.67** | **15.23** | | **15.06** | **12.98** | **12.67** |
| **MGI:1921765** | **Cdkal1** | **HMGU** | **12.94** | **13.59** | **12.30** | **12.11** | **13.75** | | **15.19** | **12.00** | **13.02** |
