# Supplementary material for: High-throughput discovery of genetic determinants of circadian misalignment
Source: PLoS Genet. 2020 Jan 13;16(1):e1008577. doi: 10.1371/journal.pgen.1008577 (PMC6980734; doi:10.1371/journal.pgen.1008577)
Supplement: S10 Table — (DOCX) [file pgen.1008577.s014.docx]

**S10 Table. Mutant lines for the secondary criteria**

| **Center** | **gene ID** | **gene Symbol** | **phenotype annotation** |
| --- | --- | --- | --- |
| **WTSI** | **MGI:1916865** | **Rab15** | **activity onset delay** |
| **WTSI** | **MGI:1919338** | **Ush1c** | **activity onset delay** |
| **WTSI** | **MGI:1920040** | **Ssbp1** | **activity phase delay** |
| **WTSI** | **MGI:1924781** | **Anks1b** | **activity phase delay** |
| **WTSI** | **MGI:106441** | **Zranb1** | **activity phase delay** |
| **WTSI** | **MGI:1920432** | **Pear1** | **activity phase delay** |
| **WTSI** | **MGI:2387643** | **B9d2** | **activity phase delay** |
| **WTSI** | **MGI:1919338** | **Ush1c** | **activity phase delay** |
| **WTSI** | **MGI:104510** | **Myo7a** | **activity phase delay** |
| **WTSI** | **MGI:104967** | **Glg1** | **feeding onset delay** |
| **WTSI** | **MGI:104807** | **Pls3** | **feeding onset delay** |
| **WTSI** | **MGI:97401** | **Ocm** | **feeding onset delay** |
| **WTSI** | **MGI:1345964** | **Coro1c** | **feeding onset delay** |
| **WTSI** | **MGI:102848** | **Serpina3c** | **feeding onset delay** |
| **WTSI** | **MGI:1351345** | **Grm8** | **feeding onset delay** |
| **WTSI** | **MGI:1920432** | **Pear1** | **feeding onset delay** |
| **WTSI** | **MGI:1927665** | **Sirt3** | **feeding phase advance** |
| **WTSI** | **MGI:1860086** | **Crlf3** | **feeding phase advance** |
| **WTSI** | **MGI:2687005** | **Leprot** | **feeding phase advance** |
| **WTSI** | **MGI:2442040** | **G3bp2** | **feeding phase advance** |
| **WTSI** | **MGI:2446249** | **Edc4** | **feeding phase delay** |
| **WTSI** | **MGI:2443584** | **L3mbtl2** | **feeding phase delay** |
| **WTSI** | **MGI:3612340** | **Ehbp1l1** | **feeding phase delay** |
| **WTSI** | **MGI:104807** | **Pls3** | **feeding phase delay** |
| **WTSI** | **MGI:2152450** | **Usp3** | **feeding phase delay** |
| **WTSI** | **MGI:1920040** | **Ssbp1** | **feeding phase delay** |
| **WTSI** | **MGI:2140680** | **A430005L14Rik** | **feeding phase delay** |
| **ICS** | **MGI:106441** | **Zranb1** | **activity onset advance** |
| **ICS** | **MGI:108017** | **Laptm4a** | **activity onset advance** |
| **ICS** | **MGI:1194495** | **Fpr3** | **activity onset advance** |
| **ICS** | **MGI:1347355** | **Slc7a11** | **activity onset advance** |
| **ICS** | **MGI:1859217** | **Dnal4** | **activity onset advance** |
| **ICS** | **MGI:1915299** | **Tgif2** | **activity onset advance** |
| **ICS** | **MGI:1918180** | **Nol8** | **activity onset advance** |
| **ICS** | **MGI:1919792** | **Pgam5** | **activity onset advance** |
| **ICS** | **MGI:1921256** | **Dnm1l** | **activity onset advance** |
| **ICS** | **MGI:1931838** | **Dbn1** | **activity onset advance** |
| **ICS** | **MGI:2147616** | **Otub1** | **activity onset advance** |
| **ICS** | **MGI:88562** | **Ctsd** | **activity onset advance** |
| **ICS** | **MGI:97511** | **Pcsk1** | **activity onset advance** |
| **ICS** | **MGI:1914430** | **Yipf5** | **activity onset delay** |
| **ICS** | **MGI:107563** | **Nab2** | **activity onset delay** |
| **ICS** | **MGI:2147616** | **Otub1** | **activity phase advance** |
| **ICS** | **MGI:1915299** | **Tgif2** | **activity phase advance** |
| **ICS** | **MGI:1923930** | **5330417C22Rik** | **activity phase advance** |
| **ICS** | **MGI:1276575** | **Ptdss1** | **activity phase delay** |
| **ICS** | **MGI:97488** | **Pax4** | **feeding phase advance** |
| **ICS** | **MGI:97381** | **Ntf5** | **feeding phase delay** |
| **ICS** | **MGI:1859162** | **Rnf10** | **feeding phase delay** |
| **ICS** | **MGI:1922863** | **Med25** | **feeding onset advance** |
| **ICS** | **MGI:1298232** | **Gzmk** | **feeding onset advance** |
| **ICS** | **MGI:97381** | **Ntf5** | **feeding onset delay** |
| **ICS** | **MGI:2652819** | **Baiap2l2** | **feeding onset delay** |
| **RBRC** | **MGI:109523** | **Trpc6** | **activity onset advance** |
| **RBRC** | **MGI:1336993** | **Ap4e1** | **activity onset advance** |
| **RBRC** | **MGI:109147** | **Oxtr** | **activity onset delay** |
| **RBRC** | **MGI:91842** | **D1Pas1** | **activity phase delay** |
| **RBRC** | **MGI:1926245** | **Ube2j1** | **activity phase delay** |
| **RBRC** | **MGI:109147** | **Oxtr** | **feeding phase advance** |
| **RBRC** | **MGI:1336993** | **Ap4e1** | **feeding phase delay** |
| **RBRC** | **MGI:109523** | **Trpc6** | **feeding onset advance** |
| **RBRC** | **MGI:109147** | **Oxtr** | **feeding onset advance** |
| **RBRC** | **MGI:1336993** | **Ap4e1** | **feeding onset delay** |
| **RBRC** | **MGI:1859162** | **Rnf10** | **feeding onset delay** |
| **TCP** | **MGI:1338944** | **Acvr1b** | **activity onset delay** |
| **TCP** | **MGI:2384891** | **Rhbdl1** | **activity onset delay** |
| **TCP** | **MGI:1343085** | **Spop** | **activity onset delay** |
| **TCP** | **MGI:3693832** | **Aktip** | **activity phase advance** |
| **TCP** | **MGI:1194508** | **Ddost** | **activity phase delay** |
| **TCP** | **MGI:2667185** | **Myo18a** | **activity phase delay** |
| **TCP** | **MGI:109573** | **Tep1** | **activity phase delay** |
| **TCP** | **MGI:96413** | **Idh1** | **activity phase delay** |
| **TCP** | **MGI:2685530** | **Gm684** | **feeding phase advance** |
| **TCP** | **MGI:1203729** | **Pik3c2a** | **feeding phase advance** |
| **TCP** | **MGI:2142527** | **Exoc8** | **feeding phase delay** |
| **TCP** | **MGI:1343085** | **Spop** | **feeding phase delay** |
| **TCP** | **MGI:1913711** | **Ptpmt1** | **feeding onset advance** |
| **TCP** | **MGI:88555** | **Ctla2b** | **feeding onset advance** |
| **TCP** | **MGI:2667185** | **Myo18a** | **feeding onset delay** |
| **TCP** | **MGI:2385191** | **Rhbdl1** | **feeding onset delay** |
| **HMGU** | **MGI:1916214** | **Ctc1** | **activity onset delay** |
| **HMGU** | **MGI:103300** | **Rbl1** | **activity phase delay** |
| **HMGU** | **MGI:98483** | **Tap1** | **activity phase delay** |
| **HMGU** | **MGI:107379** | **Gstt1** | **activity phase delay** |
| **HMGU** | **MGI:2152453** | **Gsk3a** | **activity phase delay** |
| **HMGU** | **MGI:2144041** | **Wsb2** | **activity phase delay** |
| **HMGU** | **MGI:1916214** | **Ctc1** | **activity phase delay** |
| **HMGU** | **MGI:1919912** | **Dis3** | **feeding onset advance** |
| **HMGU** | **MGI:102849** | **Klkb1** | **feeding onset advance** |
| **HMGU** | **MGI:1924182** | **Arfip2** | **feeding onset advance** |
| **HMGU** | **MGI:1351628** | **Rps26** | **feeding onset advance** |
| **HMGU** | **MGI:106181** | **Mybbp1a** | **feeding onset advance** |
| **HMGU** | **MGI:1920524** | **Gpbp1** | **feeding onset delay** |
| **HMGU** | **MGI:109211** | **Pkn2** | **feeding onset delay** |
| **HMGU** | **MGI:3505689** | **Cdsn** | **feeding onset delay** |
| **HMGU** | **MGI:98038** | **Rpl32** | **feeding onset delay** |
| **HMGU** | **MGI:95634** | **Gad2** | **feeding onset delay** |
| **HMGU** | **MGI:1347352** | **Hunk** | **feeding onset delay** |
| **HMGU** | **MGI:102849** | **Klkb1** | **feeding phase advance** |
| **HMGU** | **MGI:97489** | **Pax5** | **feeding phase advance** |
| **HMGU** | **MGI:2144837** | **Ptpn23** | **feeding phase advance** |
| **HMGU** | **MGI:1923714** | **Casc5** | **feeding phase advance** |
| **HMGU** | **MGI:99515** | **Syk** | **feeding phase advance** |
| **HMGU** | **MGI:1916658** | **Dnajc17** | **feeding phase advance** |
| **HMGU** | **MGI:95634** | **Gad2** | **feeding phase delay** |
| **HMGU** | **MGI:98483** | **Tap1** | **feeding phase delay** |
| **HMGU** | **MGI:1928483** | **Stx5a** | **feeding phase delay** |
| **HMGU** | **MGI:104897** | **Rest** | **feeding phase delay** |
| **HMGU** | **MGI:1916216** | **Ngdn** | **feeding phase delay** |
| **HMGU** | **MGI:1916214** | **Ctc1** | **feeding phase delay** |
