# Supplementary material for: High-throughput discovery of genetic determinants of circadian misalignment
Source: PLoS Genet. 2020 Jan 13;16(1):e1008577. doi: 10.1371/journal.pgen.1008577 (PMC6980734; doi:10.1371/journal.pgen.1008577)
Supplement: S11 Table — (DOCX) [file pgen.1008577.s015.docx]

**S11 Table. Hits from secondary criteria**

|  | **Genotype** | **T_on_** | **T_ph_** | ***d_on_*** | ***p_on_*** | ***d_ph_*** | ***p_ph_*** |
| --- | --- | --- | --- | --- | --- | --- | --- |
| **Activity** | ***Slc7a11^tm1b/tm1b^*** | **10.75±0.89** | **13.00±1.20** | **3.47** | **1.22E-19** | **0.05** | **0.89** |
|  | ***Oxtr^tm1.1/tm1.1^*** | **14.29±1.11** | **15.29±1.38** | **0.79** | **3.9E-2** | **0.13** | **0.73** |
|  | ***Rhbdl1^+/tm1.1^*** | **14.38±2.39** | **17.00±2.98** | **2.39** | **1.45E-9** | **0.85** | **2.5E-2** |
|  | ***Spop^+/tm1b^*** | **13.80±2.62** | **16.00±2.71** | **1.45** | **2.26E-5** | **0.30** | **0.36** |
|  | ***Ctc1^+/tm1b^*** | **13.30±1.16** | **16.00±1.24** | **5.37** | **1.20E-56** | **1.11** | **5.1E-4** |
| **Food**  **intake** | ***Slc7a11^tm1b/tm1b^*** | **11.71±1.25** | **14.00±0.93** | **0.73** | **5.6E-2** | **0.41** | **0.25** |
|  | ***Oxtr^tm1.1/tm1.1^*** | **11.20±2.28** | **13.8±2.28** | **1.97** | **1.95E-05** | **0.68** | **0.13** |
|  | ***Rhbdl1^+/tm1.1^*** | **13.17±2.79** | **15.33±2.94** | **0.13** | **0.75** | **0.32** | **0.45** |
|  | ***Spop^+/tm1b^*** | **16.11±2.57** | **17.56±2.70** | **3.89** | **1.05E-23** | **1.15** | **1.5E-3** |
|  | ***Ctc1^+/tm1b^*** | **14.30±2.31** | **16.60±1.78** | **1.71** | **8.04E-8** | **0.25** | **0.43** |

**T_on_: onset time**

**T_ph_: peak phase**

***d_on_*: effect size for onset times**

***p_on_*: p value of student’s test of wild type and mutants’ onset times**

***d_ph_*: effect size for peak phases**

***p_ph_*: p value of student’s test of wild type and mutant’s peak phases**
