# Supplementary material for: High-throughput discovery of genetic determinants of circadian misalignment
Source: PLoS Genet. 2020 Jan 13;16(1):e1008577. doi: 10.1371/journal.pgen.1008577 (PMC6980734; doi:10.1371/journal.pgen.1008577)
Supplement: S12 Table — (DOCX) [file pgen.1008577.s016.docx]

**S12 Table. Phenotype associated assay**

| **Genotype** | **Phenotypes** | **p value** |
| --- | --- | --- |
| ***Rhbdl1^+/tm1.1^*** | **Glucose Tolerance** | **0.00075** |
| ***Slc7a11^tm1b/tm1b^*** | **Glucose Tolerance** | **0.00080** |
