# Supplementary figures and images for: High-throughput discovery of genetic determinants of circadian misalignment
Source: PLoS Genet. 2020 Jan 13;16(1):e1008577. doi: 10.1371/journal.pgen.1008577 (PMC6980734; doi:10.1371/journal.pgen.1008577)

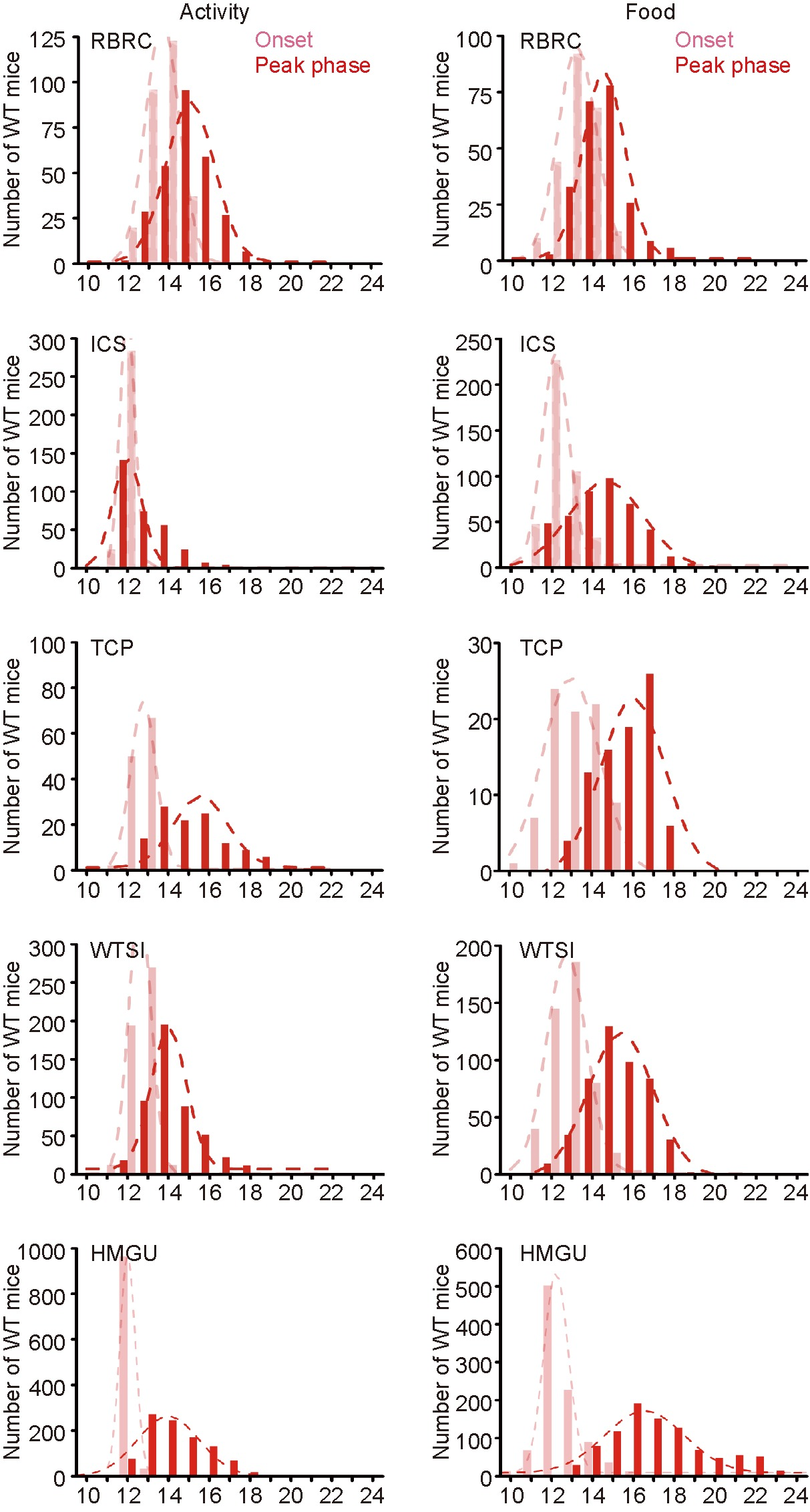

Supplement: S1 Fig — Histogram of the onset and peak phase results obtained from five IMPC centres (ICS, WTSI, RBRC, TCP and HMGU) under 12-hour light and 12-hour dark cycles. n = 2201 C57BL/6N mice for activity and n = 2160 C57BL/6N mice for food intake measured by indirect calorimetry over time. Pink column: onset time, red column: peak phase. (TIF) [file pgen.1008577.s017.tif]

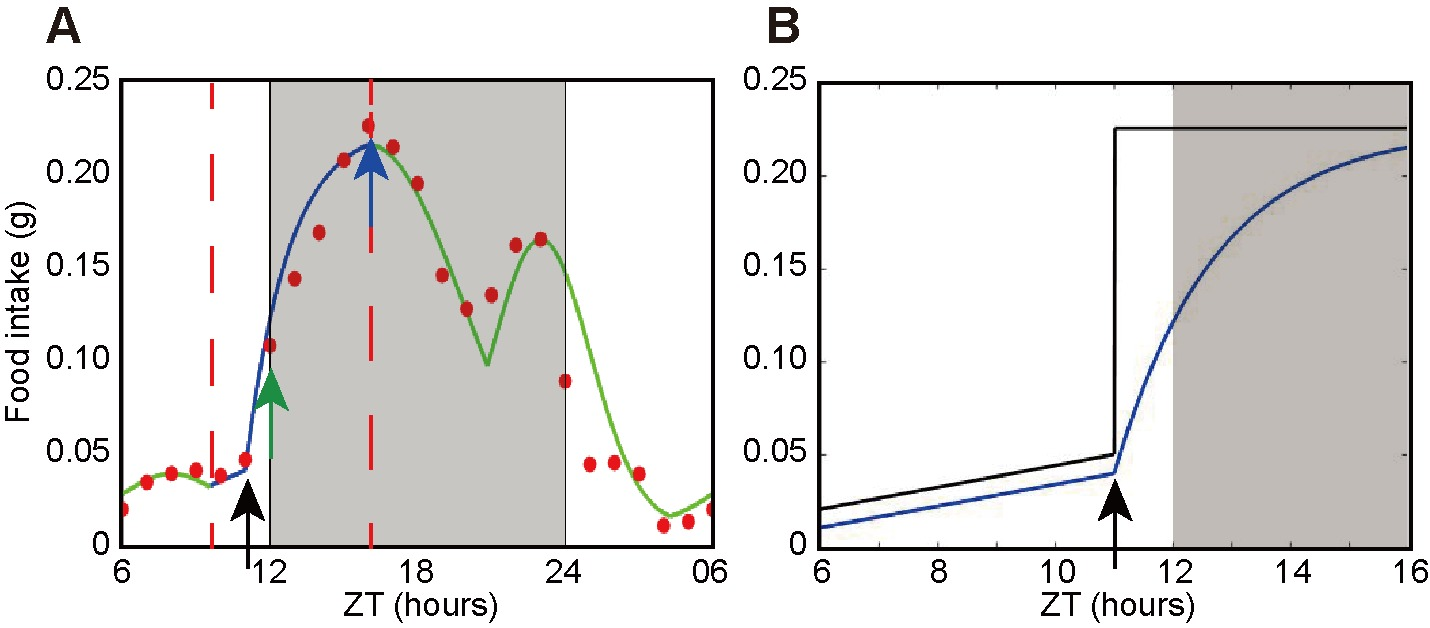

Supplement: S2 Fig — (A) Mean data of food intake for wild-type mice from HMGU as an example. Red dots are averaged raw data and multicoloured curve is the fitted curve. Part data before peak phase (data between two red dashed lines) are fitted by a piecewise function in (B).Blue curve is the fitting curve. Onset is identified as the first data entering transition (green arrow), while peak phase is at the end of transition (blue arrow). Other data in (A) are fitted by Gaussian functions (green curves). (B) Piecewise function for fitting data points before E peak phase. Dividing points of two stages are indicated by two black arrows in (A) and (B). (TIF) [file pgen.1008577.s018.tif]

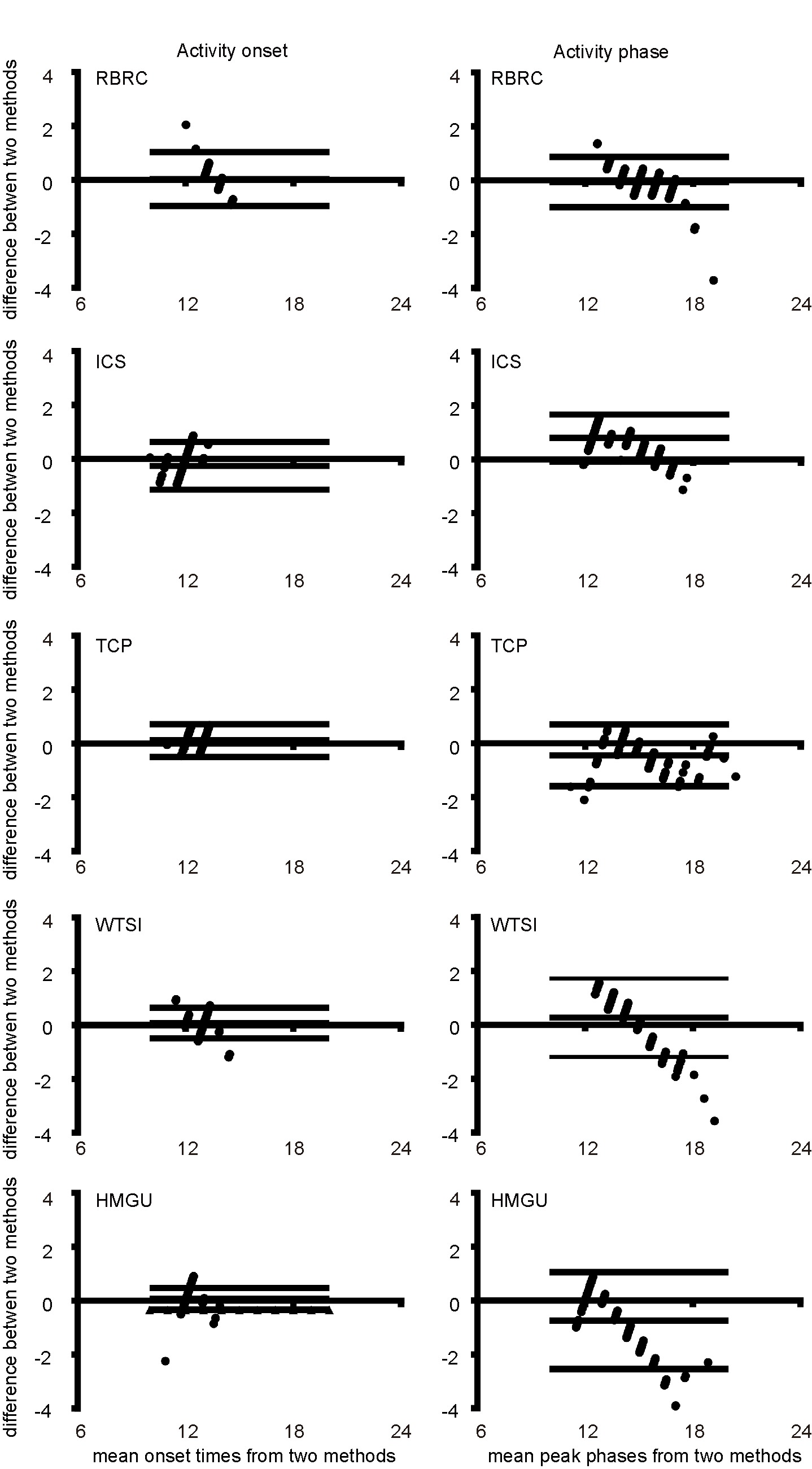

Supplement: S3 Fig — The 95% limits of agreement (1.96 s.d.) were calculated to determine whether the SyncScreener could replace visual assessment. Activity onset data and peak phase activity data obtained by the five centres (ICS, WTSI, RBRC, TCP and HMGU). (TIF) [file pgen.1008577.s019.tif]

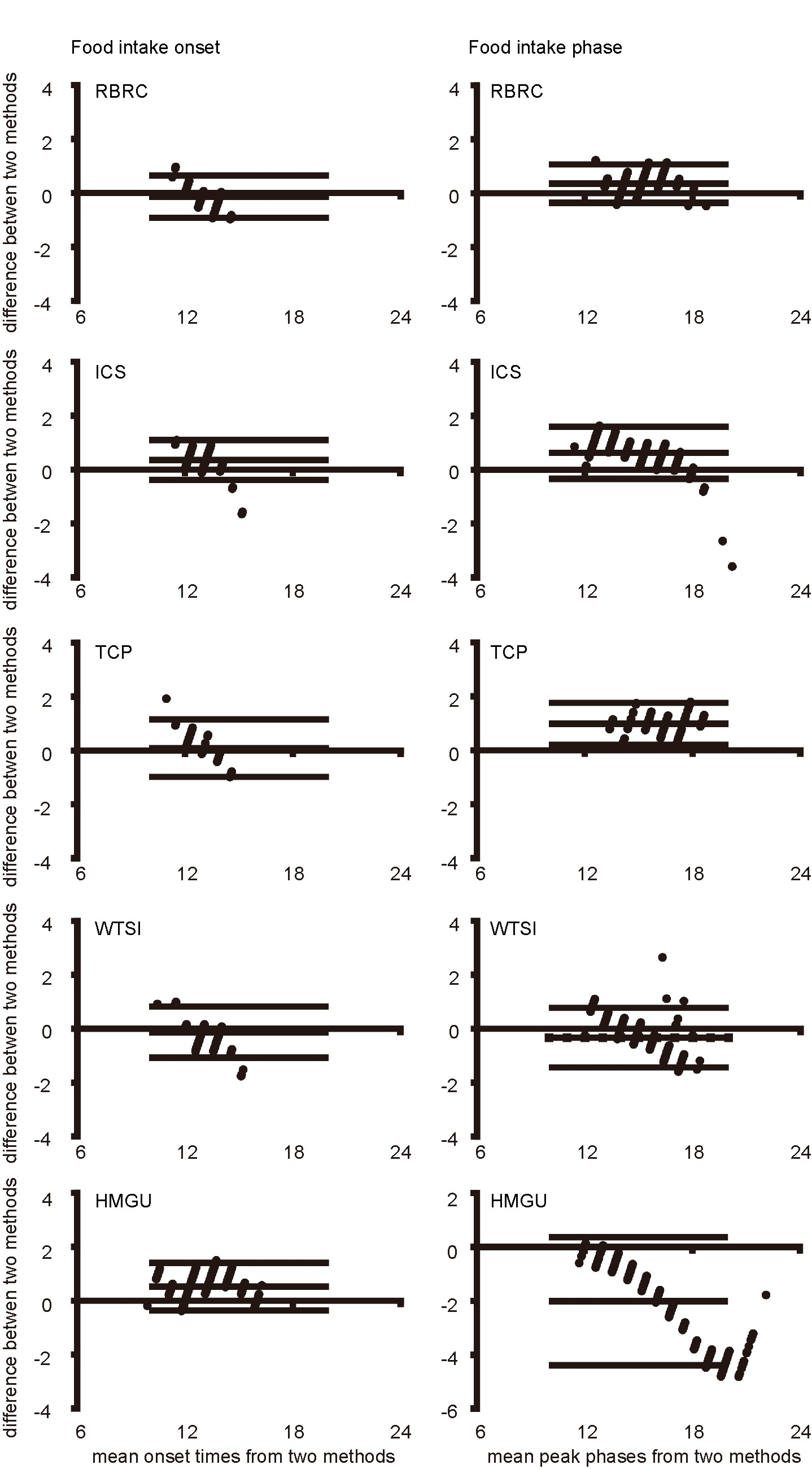

Supplement: S4 Fig — Food intake onset data and peak phase data obtained by the five centres (ICS, WTSI, RBRC, TCP and HMGU). (TIF) [file pgen.1008577.s020.tif]

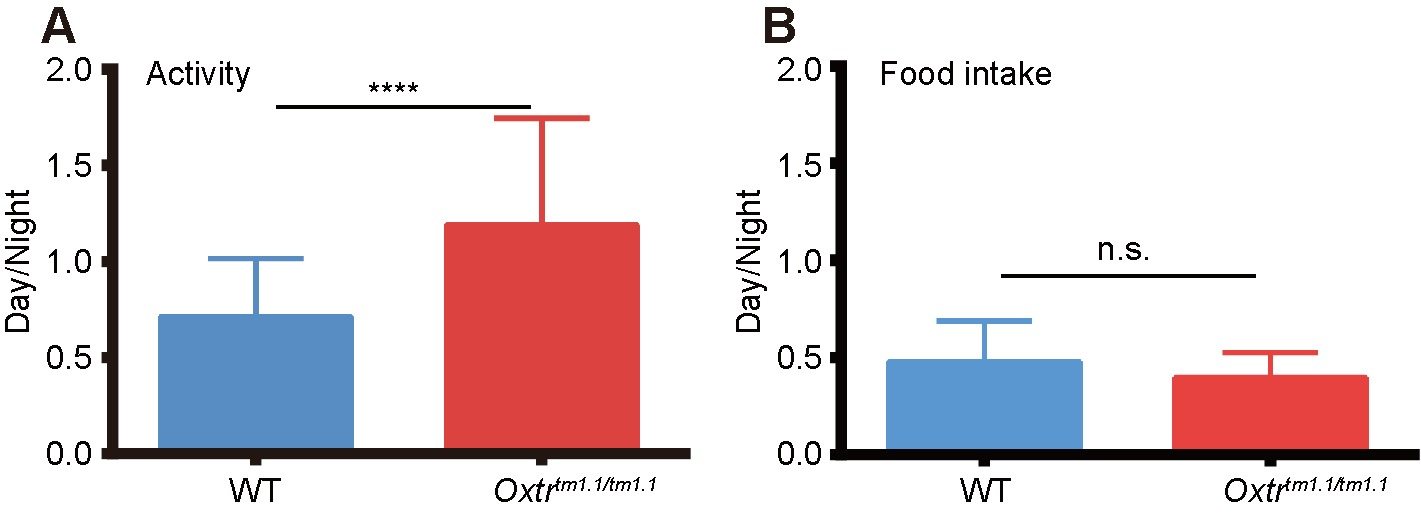

Supplement: S5 Fig — (A and B) Ratios of daytime activity (A) and food intake (B) to that at night were calculated using data from Oxtrtm1.1/tm1.1 and wild-type mice data from RBRC. Two-way ANOVA was employed to test the statistical significance. ****: P < 0.0001. (TIF) [file pgen.1008577.s021.tif]

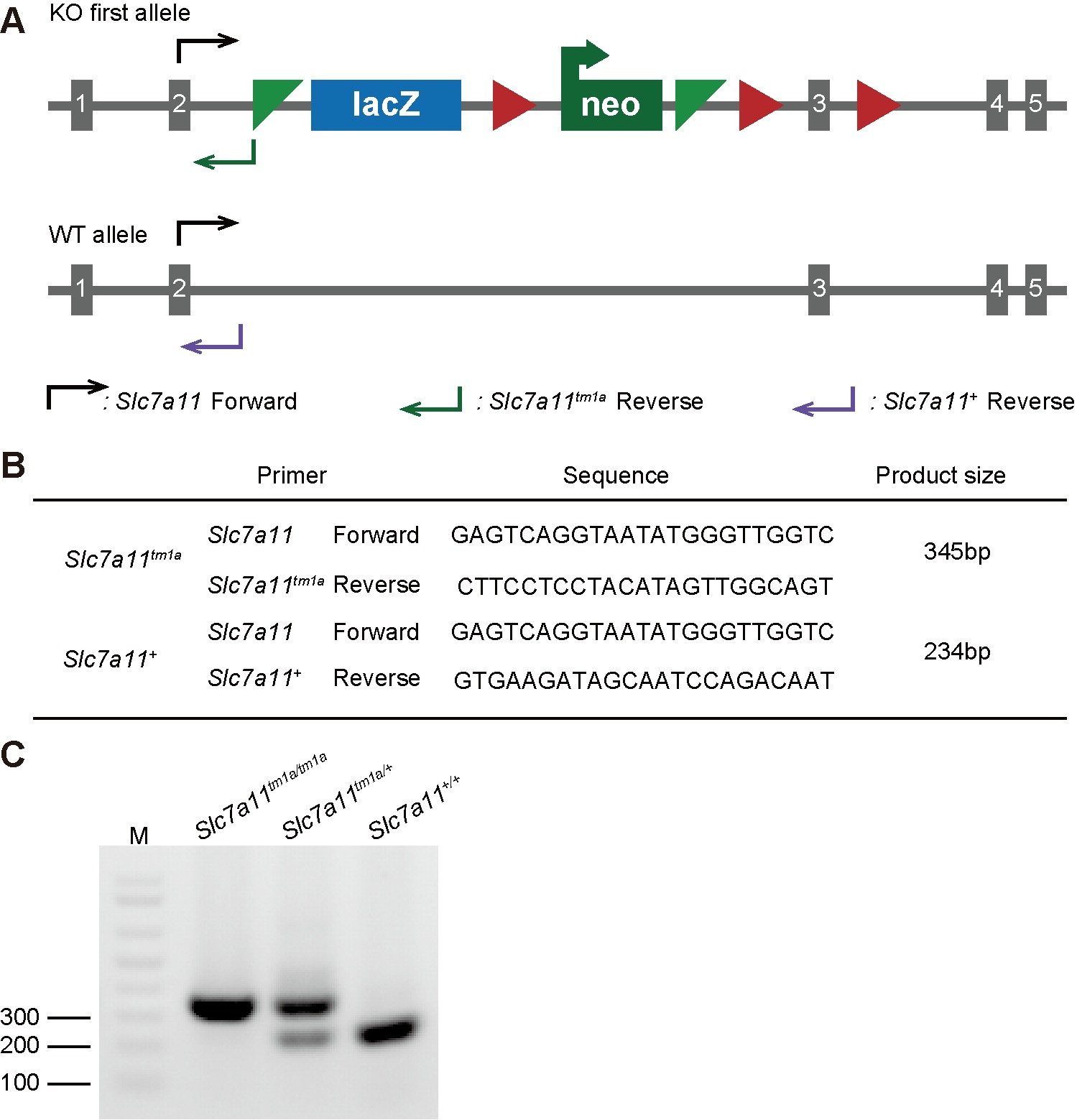

Supplement: S6 Fig — (A) Schematic of knockout strategy for Slc7a11 based on knockout-first design. (B) Forward and reverse primers for genotyping. (C) PCR analysis of tail genomic DNA for wild-type and Slc7a11tm1a/tm1a alleles in wild-type, heterozygous and homozygous knockout mice. (TIF) [file pgen.1008577.s022.tif]

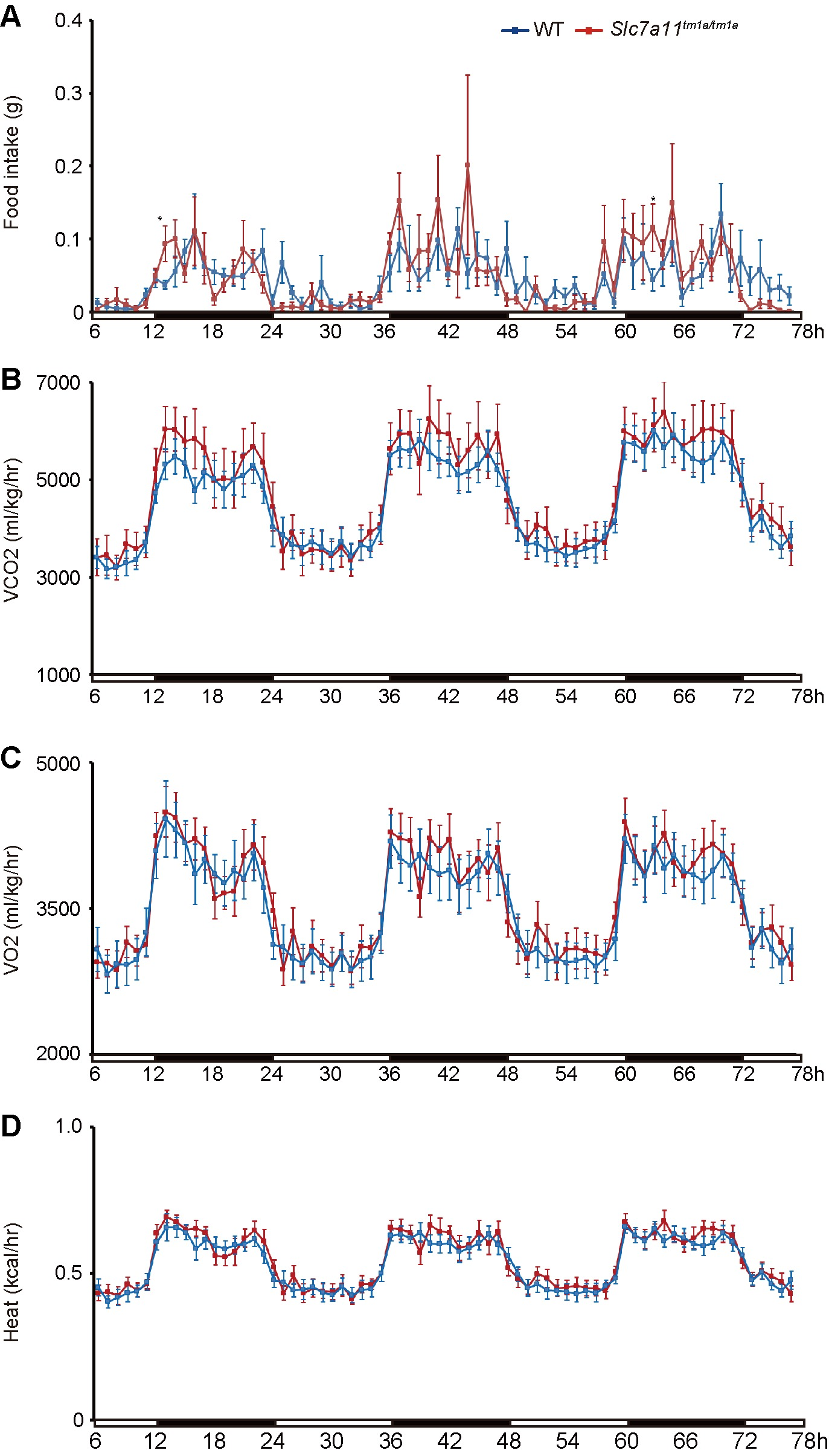

Supplement: S7 Fig — (A) food intake; (B) the volume of CO2; (C) the volume of O2; (D) the heat. Rhythms of food intake, VCO2, VO2 and the heat were plotted over a 72 hr time frame as the mean ± SEM (n = 10). Two-way ANOVA was used to determine the statistical significance, *p < 0.05. (TIF) [file pgen.1008577.s023.tif]

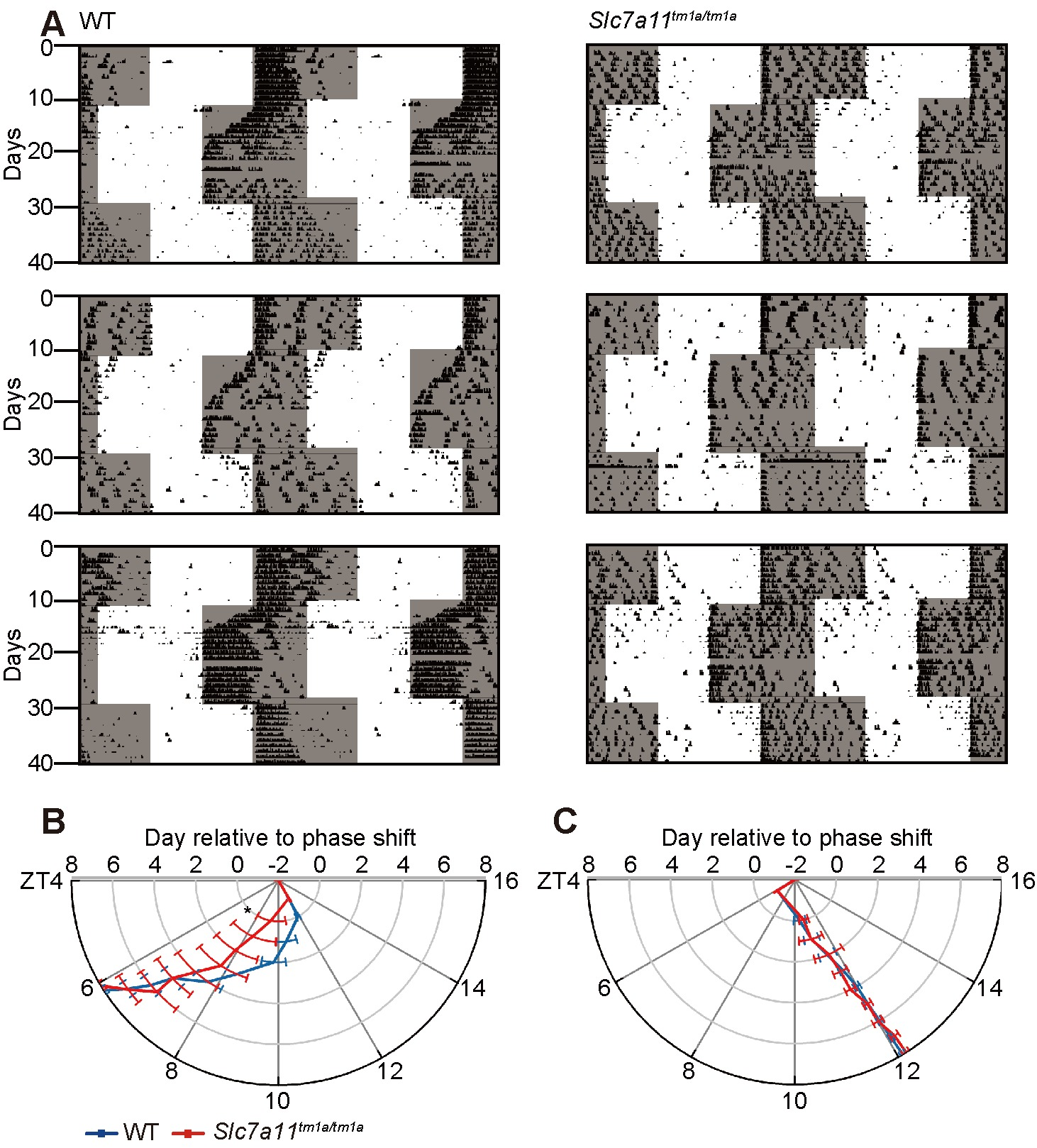

Supplement: S8 Fig — (A) Representative actograms of wheel-running activity of wild-type and Slc7a11tm1a/tm1a mice subjected to a 6-hr phase advance and delay in LD cycle. At day 22, the recording was disrupted for about 24 hours. (B and C) Re-entrainment traces of phase advance (B) and delay (C) of wild-type (Blue) and Slc7a11tm1a/tm1a (Red) mice. n = 9 for wild-type mice, n = 5 for Slc7a11tm1a/tm1a mice. Two-way ANOVA was employed to test the statistical significance. *: P < 0.05. (TIF) [file pgen.1008577.s024.tif]

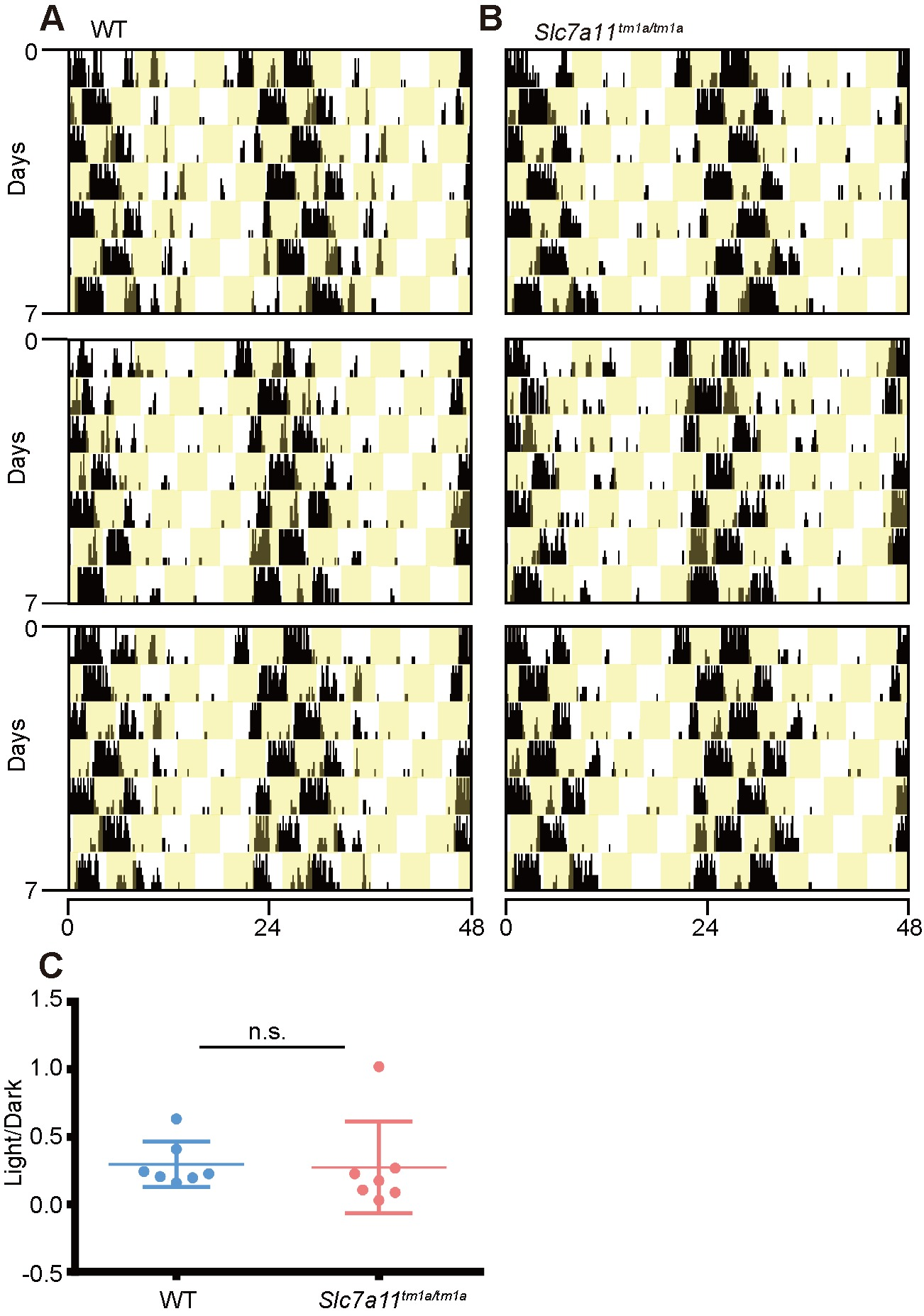

Supplement: S9 Fig — (A and B) Representative actograms of daily wheel-running activity of wild-type (A) and Slc7a11tm1a/tm1a mice (B). Light phases are indicated in yellow to show the structure of the LD 3.5:3.5 cycle as well as to help visualize the occurrence of wheel-running activity under this schedule. (C) Masking ratios of wild-type and Slc7a11tm1a/tm1a mice during LD 3.5:3.5, which are calculated by dividing total activity during light phases with that during dark phases. n = 7 for each genotype. Two-way ANOVA was employed to test the statistical significance. n.s.: P >0.05. (TIF) [file pgen.1008577.s025.tif]

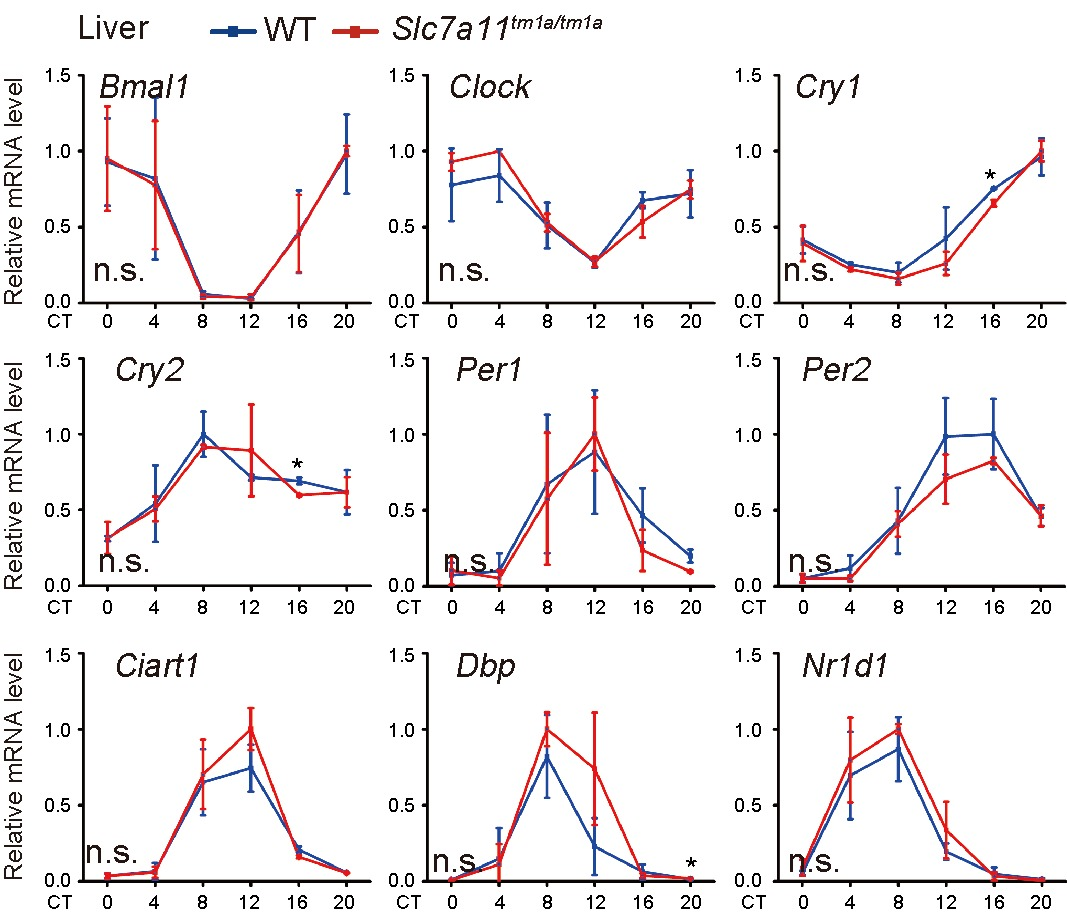

Supplement: S10 Fig — Error bars represent the s.d. for each time point from three biological independent replicates. Two-way ANOVA was employed to test the statistical significance. n.s.: P >0.05. (TIF) [file pgen.1008577.s026.tif]
