# Supplementary material for: High-throughput discovery of genetic determinants of circadian misalignment
Source: PLoS Genet. 2020 Jan 13;16(1):e1008577. doi: 10.1371/journal.pgen.1008577 (PMC6980734; doi:10.1371/journal.pgen.1008577)
Supplement: Attachment — Submitted filename: Responses 20191108FF.docx [file pgen.1008577.s027.docx]

Dear Achim,

Thank you very much for your reply and for giving us the chance to revise our manuscript according to the reviewers’ and your constructive comments. We are pleased that you and the reviewers consider our study of sufficient interest for possible publication in PLOS Genetics. I will briefly address the principal issues raised by the reviewers and then a detailed point-by-point response to all comments (reviewers’ comments in blue, our responses in black).

1. We have added a simulated Jet lag, Photoperiod and T cycles experiments in the revised manuscript (Fig. 6H-J, S8 Fig and S9 Fig). We also examined the high-amplitude expression genes such as *Dbp*, *Ciart*, *Nr1d1* in the revised Fig 7D, showing a subtle but advanced expression phase. These results further validated our screening and supported our conclusion that *Slc7a11* is involved in the circadian entrainment.
2. Other points. (1) We have added Fig 2E-H to clarify the variety of the screening parameters and given more information in the revised Fig 1. (2) We have provided all activity and food intake data for those positive controls in the revised Fig 3A-J, and provided their parameter value and statistical value and effective size in the revised Fig 3K. (3) We added metabolic cage data for food intake VCO_2_, VO_2_, and heart in the revised S7 Fig. (4) We also added activity ratios of day time and night time for *Oxtr^tm1a/tm1a^* mice in the revised S5 Fig.
3. Fig 4 had been redrawn. We also provided graphical explanation for onset determination in S2 Fig.
4. We have also corrected other issued raised by the reviewers, which we hope satisfies the requirements for publication in PloS Genetics.

**Reviewer #1**

Zhang et al. developed a high-throughput screening approach to identify genes involved in circadian entrainment in mice. They use WT activity and feeding data from five different IMPC centers as reference data set and test this against data from 750 mutant lines. They identify 5 new mutants of which one is followed up. Slc7a11 mutant mice show advanced activity onsets and some changes in the expression of cell coupling genes in the SCN. The paper describes an interesting and very promising approach - and a clever use of existing consortium data. I have two major points of criticism:

Q1. Maybe I misunderstood this, but to me it is somewhat surprising that from the 750 screened lines no known clock regulators with activity phenotypes have emerged.

**Response:** We thank the reviewer for raising this issue. We have previously searched the known clock regulators on locomotor, entrainment or output from 750 screened lines and found that only *Rora* and *Jmjd1c* are potential candidates for effects on locomotor activity. Since *Rora* and *Jmjd1c* homozygous mice are subviable, we’re only able to check IC data from heterozygous mice and did not find significant differences. Therefore, we established positive controls using several known circadian mutant lines (*hPER2^S662G^*, *Fbxl3*^-/-^ , *Nestin-Cre:Zbtb20*^-/-^ and *hPER1^S714G^*) with activity or food intake phenotypes. To address this concern, we added a sentence to explain why we needed to use the positive controls (p 11 line 232-234): “since we did not find known clock regulators with activity phenotypes from 750 screened lines, we used several known circadian mutant lines to validate our predictive models and parameters”.

Q2. While the proclaimed target of the screen is circadian entrainment only LD12:12 activity and gene expression data are shown, and no entrainment-targeted experiments were performed.

**Response:** Thanks for bringing this point up. Together with reviewer3’s point, we added circadian entrainment-targeted experiments. We performed skeleton photoperiod and simulated jetlag (advance) experiments. Interestingly, in response to a 6 h advance shift of the LD cycle, *Slc7a11^tm1a/tm1a^* mice immediately showed phase advance (Revised S8 Fig). Furthermore, to minimize the masking effect of light, *Slc7a11^tm1a/tm1a^* mice were subjected to a skeleton photoperiod with 15-min light arms from clock time 07:45 to 08:00 and clock time 20:00 to 20:15 (light on 08:00) with darkness at all other times. We found that the activity onset was significantly advanced in *Slc7a11^tm1a/tm1a^* mice compared with wild-type littermates (Revised Fig 6H-J). These results would enhance our conclusion. As reviewer 3 also pointed out, we acknowledge that we misused “entrainment” in the manuscript especially in the screen part. It would be better to say “misalignment” instead of “entrainment”. We have rephrased these sentences to clarify this point in the revised manuscript.

Further points:
Q3 It is surprising that the activity phase advance for the Slc7a11 mutants is rather mild and not very robust. Was this one of the best mutants? How often were strong onset advances or delays (+/- 3 hrs or more) observed?

**Response:** We thank the reviewer for pointing this out. There’re two reasons for us to choose *Slc7a11* to validate our screen methods. First, *Rhbdl1*, *Spop* and *Ctc1* homozygous mice were preweaning or embryonic lethal, which made it impossible to perform further circadian experiments using homozygous knockout. Thus, we generated *Slc7a11* and *Oxtr* mutant mice (onset advances). In *Oxtr^tm1a/tm1a^* mice, we did observe a behavioral abnormality. However, due to lactation problems and lack of maternal love of female, we don’t have enough mice to do circadian entrainment experiment. We hope this finding will become another new project. Compared to phase delays, the phase advance is thought to be much less common but more meaningful. We found only one line from 750 mutant lines. *Slc7a11* mutant mice showed a 1-hr advance milder than *hPER2^S662G^* (1.65-hr advance) but stronger than in *hCRY2A260* mutant mice (0.5-hr advance), which have advanced sleep phase syndrome (Hirano A et al, 2016).

Q4 Line 103 – would suggest inserting “but may also promote” before “sleep disorders” as chronodisruption is not the only cause for the following disorders.

**Response:** Thank reviewer’s suggestion. The sentence has been changed.

Q5 Line 147 – make sure to clearly distinguish between “circadian” and “diurnal”. Since you are studying LD conditions in most cases, “daily” or “diurnal” would be correct here.

**Response:** We apologize for this mistake. The term has been rectified in the revised manuscript.

Q6 Page 9 – why were activity data binned at 1-h intervals? For the machine learning algorithm this should not make much of a difference and you are simplifying the data, thus potentially masking interesting phenotypes.

**Response:** We thank the reviewer for the insightful comments. This is true that lowing sampling resolution could result in missing interesting phenotypes and that it does not make much of a difference when using machine learning algorithms. However, we performed data binning primarily because sampling intervals were not identical among the five IMPC centers, (RBRC: around 1 hour, TCP: around 20minutes, ICS: 1hour, WTSI: 13, 15, 20, 22, 23, 26 minutes, etc, HMGU: around 15minutes). Therefore, we binned data at 1-h interval to unify data format, which made it possible to apply the same deep learning neural networks to generate predictive models. Unifying data format also makes the results from different IMPC centers comparable with the same sampling rate.

Q7 Line 193 – you argue that you excluded M peaks from analysis because they are less robust, but what would that mean for entrainment? Entrainment phase should be reflected in both peaks. I guess there is a bit of a misconception between “entrainment” and “activity pattern/profile” throughout the paper. Along this line it does not surprise that SCN clock gene expression is not consistently altered in the Slc7a11 mutant since effects could be downstream of the SCN.

**Response:** As acknowledged by both reviewers #1 and #3, “entrainment” is not a suitable term in our paper, we have modified “circadian entrainment” to “circadian misalignment” in the revised manuscript including the title. Based on our results, onset is more robust than offset. Since M peak is lower than E peak and its dispersion of M peak is greater. We thus excluded M peaks from the analysis. In addition, **a** previous study revealed that M peak and E peak phase are not synergistic: in Drosophila, LNvs is sufficient to restore the morning activity peak but not the evening activity (Grima et al., 2004), suggesting that M peak may be a relatively independent oscillator different from E peak.

As suggested in Q12 by reviewer, we examined high-amplitude gene expression profiles of *Dbp*, *Ciart* and *Nr1d1*. Overall, the peaks of *Dbp* and *Per2* expressions were advanced. This result is also consistent with the advanced activity. We have added new results in revised Figure 7D.

Q8 Line 265 – what do you mean by “similar phenotypes”?

**Response:** We appreciate this question from the reviewer and regret not explaining this word clearly in the manuscript. Similar phenotypes mean that phenotypes of individual mutant mice are similar to that of average data (7 mice). Phenotyping of 7 male or 7 female mutant mice were investigated following the adult pipeline of IMPC including indirect calorimetry. Since not all mice of a mutant have similar phenotypes as a result of incomplete penetrance (may due to cell-autonomous, stochastic variation in gene expression in components of disrupted buffer pathway), the IMPC protocol requires phenotyping baseline for large scale screening. In our study, 50% of individual mice within a line displayed similar phenotypes as one of phenotyping baseline as described previously (de Angelis et al., 2015; Dickinson et al., 2016), together with effect size, p value, we obtained potential targets like to other IMPC screening protocol.

Q9 Page 14 – to assess entrainment I would expect T-cycle experiments or experiments under different light intensity conditions. Could the advances of activity onsets in the Slc7a11 mutants be explained by altered light masking? Do the mice entrain faster/slower under shifted LD cycle conditions?

**Response:** As suggested by reviewer #1 and #3, we have now added skeleton photoperiod and Jetlag, as shown in the revised Fig 6I-6K, and S8 Fig, in response to a 6 h advance shift under the LD cycle *Slc7a11^tm1a/tm1a^* mice immediately showed phase advance and during skeleton photoperiod stimuli under constant darkness, *Slc7a11^tm1a/tm1a^* mice showed phase advances. Furthermore, mice were also exposed to an alter cycle of 3.5 h light and 3.5 h dark for T-cycle experiments as suggested. *Slc7a11^tm1a/tm1a^* confined their activity mostly to the dark phase as their littermates did in the revised S9 Fig, suggesting that disruption of *Slc7a11* did not affect masking behavior. Therefore, we did not further change light intensity. We very much appreciate the reviewer’s suggestion, as we feel now that these results do even strengthen our conclusion.

Q10 Figs. 3 & 5 – relabel “ZT30” to “ZT6”

**Response:** We thank the reviewer for raising this point. We have relabeled Figs.3 and Figs.5.

Q11 Fig. 7C – why was gene expression not tested in the SCN? How were CTs determined for arrhythmic Per1/2 and Bmal1 mutants? It is very counterintuitive that Slc7a11 gains rhythmicity in the two clock-less mutants compared to WT animals.

**Response:** We appreciate the reviewer’s comment here. Since the SCN samples are very tiny compared to olfactory bulb, we lacked SCN stock samples from *Bmal1*^-/-^ and *Per1/2*^-/-^ mice. Although we made every effort to breed these mice, we didn’t obtain enough *Bmal1*^-/-^ and *Per1/2*^-/-^ mice to examine the expression of *Slc7a11* in the SCN. To avoid the confusion, we removed Fig 7C in the revised manuscript. In addition, we added the expression profile of *Slc7a11* in the SCN under DD to examine its rhythmicity in the revised Fig 7C.

Q12 Fig. 7D – clock gene expression appears very much dampened already in the WT animals. Why were genes with very little rhythmicity in the SCN (Clock, Cry2) tested, but not high-amplitude genes such as Dbp or Nr1d1?

**Response:** We thank the reviewer for pointing this out and regret insufficient consideration when testing clock genes. We have tested high-amplitude genes: *Ciart1*, *Dbp* and *Nr1d1* in the SCN and liver of *Slc7a11^tm1a/tm1a^* mice and wild-type littermates (Fig 7D in revised manuscript). The mRNA levels of *Ciart1*, *Dbp* and *Nr1d1* in the SCN and liver of *Slc7a11^tm1a/tm1a^* mice were comparable with wild-type littermates. However, the phases of *Per2* and *Dbp* found in SCN of *Slc7a11^tm1a/tm1a^* mice are advanced compared to wild-type littermates (Fig 7D), which is consistent with the advanced activity onset, suggesting that disruption of *Slc7a11* affects circadian phase. These results have been added to Fig 7D and S10 Fig in the revised manuscript.

Q13 Fig. 7E – this figure is confusing to me. Several of the tested genes are not rhythmic in the WT SCN. Does this mean the effect of Slc7a11 is independent of the clock? If so, can one then speak of “circadian entrainment” (see also above)?

**Response:** We appreciate the concern raised by the reviewer. We repeated the experiments several times and the patterns are very similar as presented in Fig. 7E & 7D. We reasoned that the phases of clock genes such as *Bmal1* and *Per1* varied greatly from astrocytes, ependymal cells, endothelial cells, microglia, neurons, NG2 cells to oligodendrocytes. We admitted we cannot rule out other cells during collecting the SCN samples that contribute to dampen the rhythmicity. Thanks for the reviewer’s suggestion, we examined the high-amplitude expression of *Dbp*, *Ciart*, *Nr1d1*, together with previous *Per2* gene expression. We found that the phases of these gene expressions are advanced in *Slc7a11* KO compared in WT in the revised Fig 7D. In addition, the misalignment binding time of GRP and GRPR, and altered *Vip* and *Avp* expression may weaken the circadian entrainment in *Slc7a11* mutants. Together with the results from jet-lag (revised S8 Fig) and skeleton photoperiod experiments (revised Fig 6H-J), these results help to clarify that *Slc7a11* should be responsible for circadian entrainment of behaviour.

**Reviewer #2**

Zhang et al. analyze indirect calorimetry data form IMPC centers to extract circadian entrainment data. Overall this is paper carries out good analysis of existing data to extract new phenotypes. I certainly think it deserves publication in PLOS Genetics. I have several recommendations that will improve clarity of the paper.

Q1 Major – sex is not addressed in the paper. This is a major factor that affects many diurinal/circadian parameters, particularly during estrus (https://www.ncbi.nlm.nih.gov/pmc/articles/PMC4288375/). The authors should model sex in their analysis.

**Response:** We fully understand this concern raised by the reviewer. We agree with the reviewer that there are sex differences in circadian behavior. But we only included male mice because: 1) We found that the peak phase of food intake varied greatly in females, most likely due to estrous cycles, 2) Variability in activity onset is considerably greater in females than in males, 3) IC data from WTSI only contained male data. We think that the biomarkers should be less affected by other factors including female estrous cycles. To address this concern, we have added this fact into the revised manuscript p 10 line 203-204.

Q2 The method in which mutants were identified is not clear or explained (pvalue, effect size, and variance are combined to find 5 affected mutants. The process is arbitraty.

**Response:** We appreciate raising this question and we are sorry for not explaining the process clearly in the manuscript. In the “second criteria”, we have clarified the three criteria we listed in manuscript. The three criteria: 1) 50% of individual mice within a line displayed similar phenotypes as described in a previous study (de Angelis et al., 2015; Dickinson et al., 2016; Potter et al., 2016). We applied this criterion to exclude occasional situation. 2) standardized effect size (Cohen's d), where $d=\frac{\overline{x_{m}}-\overline{x_{wt}}}{s}$ is the absolute difference between mutant and baseline means scaled in units of phenotypic standard deviation. A larger [value](https://en.wikipedia.org/wiki/Absolute_value) of effect size always indicates a stronger phenodeviance (de Angelis et al., 2015; Dickinson et al., 2016). Effect size (d) >1.2 suggested that the group difference is large between the mutant mice and the wild-type mice (<https://en.wikipedia.org/wiki/Effect_size>). 3) two-tailed t-test was used to examine the statistical significance (p value), p<0.001 means statistical significance between the mutant line and phenotyping baseline. The mutant mice which meet all three criteria are defined as potential hits for further screening. We have modified the sentence about secondary criteria in the revised manuscript (p 13, line 271 to 279) and explained the formula of effect size in material and methods (p31-32, line 652-662).

Q3 The machine learning based method developed here (SyncScreener) it is not stated how and where this will be distributed.

**Response:** We thank the reviewer for pointing out this. We will upload compressed files including code and instruction manual as supplementary files (S3 File). Also, the program is available for researchers via a personal request to Y.X.

Q4 CAM-SU Genomic Resource Center is cited as receiving S1 File and S2 File. There is no information on how the reader can access this. If this is not a maintained data repository please deposit the data at other well curated databases.

**Response:** We thank the reviewer for this suggestion. We’ve uploaded the compressed files to PLOS Genetics and these files should be available from PLOS Genetics Port. The IC data can be directly downloaded from IMPC web access. Also, pictures in S1 File and S2 File are available for researchers via cloud storage of CAM-SU Genomic Resource Center upon authorization.

Q5 Will the analysis performed here be part of the mousephenotype.org (impc) website? Given these are IMPC strains, it would be good to have this data available on this site for the community.
**Response:** All phenotyping data are accessible via the IMPC website www.mousephenotype.org. We are currently planning to implement a special web-based analysis tool as add-on to the existing stats package to account for the high complexity of indirect calorimetry data. This add-on will improve the visibility of energy expenditure, food uptake, and activity patterns. Clear links to this portal will be provided from the IMPC web portal as part of "news feature" that will promote the tool as well as from the dedicated gene pages for which the calorimetry analysis was performed.

Specific comments –
Q6 Figure 1 – I didn’t find this figure useful. It outlines the flow of the paper but does not add any information. It also uses terms that are confusing and misleading.
For instance – “Discovery of genetic determinants” is genotype association. The terms primary and secondary screen makes it seem as if there were two levels of screening. This should state something like primary criteria and secondary criteria. “Validation” was done by regenerating mutant lines for wheel running or longer IC studies. This is not conveyed in the figure. Similarly, the figure legend lacks description.

**Response**: The reviewer is quite right. We have modified the statement in Fig 1. “Discovery of genetic determinants”, “primary screen” and “secondary screen” has been modified to “Discovery of phenodeviance”, “primary criteria” and “secondary criteria”. We have re-edited Fig1 adding information related to the five centers and the steps of validation (see figure below). In addition, detailed description has been implemented in the figure legend (Fig 1). We feel that a graphical explanation will help readers to understand the logical flow and hope that the reviewer agrees.

Q7. In general, a figure outlining the process of IC at the various centers will be more useful. Emphasize the differences between the centers. Even though the protocols are available online, the paper should concisely state the detailed protocol at the centers and emphasize the differences (light cycle etc.) between the centers. If not in this figure, it should be a supplementary figure. This will help orient the reader on the challenges in analyzing this data.

**Response**: Thanks for bringing this point up. Considering the light is a major factor for circadian entrainment, we highlighted the light information in the revised Fig. 1. We also added equipment information in each center in Materials and Methods.

Q8 Minor – typo in figure. “Cellecting” should probably be “collecting”.
**Response**: We thank the reviewer for pointing the typo. We have corrected it in Fig.1.

Q9 Figure 2 –
A, B - The data is only shown for 18 hours (x-axis). The text does not clearly explain why. I’m assuming that this the general length of the IC protocol. Most reader will expect a 24 hr LD bar. HMGU is padded on the right with 0, please explain this. The data is sorted on the y axis (mice seem to be organized by peak phase), however, nothing is stated about this in the figure legend.

**Response**: Thank you for noticing this and we apologize for not explaining clearly. For light schedules and data recording intervals in each center are different, the time (x-axis) in Fig 2A and B depicts the Zeitgeber time (ZT) (ZT 0: light on, ZT12: light off). We have added “ZT” labels in Fig 2A and B with detailed description in the figure legend. Because recording started and ended at different time points in each center, we choose overlapping periods (18 hr) shown in Fig 2A and B. We are very sorry for padding 0 on the right of HMGU heat map. We have corrected it. We have added a sentence ‘mice were ordered according to their evening peak phases’ in the figure legend in p20, line 416.

Q10 Page 9, line 180 states “moreover, these two parameters exhibited a stable phase relationship…” I’m not sure what is meant by a stable phase relationship. Do you mean the phase relationship is consistent across centers or within a center or both. They certainly vary across centers – the difference between Activity peak phase and onset varies from 1 hr to 3hr. Please clarify these for the reader.

**Response**: We apologize for the confusion and thank the reviewer for pointing out. These sentences have been removed from the manuscript.

Q11 Line 197-199 describe LD conditions and are not in the methods. See earlier point about describing experimental differences between centers.
**Response**: Thank you for noticing this. As suggested, we added more information in the revised Fig. 1 including light cycles in the five centers, validation steps. We also stated the differences among centers in the Materials and Methods.

Q12 Figure 3 – why are the food intake pattens not shown for Fbxl3 and Zbtb20?

**Response**: As suggested, we have added food intake results and screen parameters in the revised Fig 3.

Q13 Which center was the validation data generated?
**Response**: Validation data were generated by Xu lab in CAM-SU Genomic Resource Center. *Fbxl3*, *Zbtb20*，PER1^S730G^ mutant mice were generated by Xu lab as described previously and PER2^S662G^ was generated by Dr. Xu in Fu & ptacek lab in the UCSF. IC data from these mice liens were generated by Xu lab in the CAM-SU Genomic Resource Center. We have clarified this in the material and methods in p23-24, line 489-497.

Q14 Figure 4 – this is a key figure that describies finding the mutants using the methods developed above. I find that a clear figure showing the distribution of the 5 final mutants is missing. I would like to see the phenotype(s) of these plotted against controls or other mutants. The “secondary screen” is actually just a secondary criteria set that the original mutants are placed through. Please explain the rationale for these – why are all lines that have a greater than 2sd effect size selected? Why not use pvalue from the onset regardless of the effect size. The rationale for comparison is not clear. Effect size is used in primary and then a combination of effect size, pvalue (strict at 0.001), and variance (50% must have significant phenotype) is used in the secondary critera. This seems like conditions were tweaked till an acceptable number of hits arose. This should be clarified.

**Response**: As suggested, *Slc7a11^tm1a/tm1a^*, *Rhbdl1^+/tm1a^*, *Spop^+/tm1a^*, *Oxtr^tm1a/tm1a^* and *Ctc1^+/tm1a^* have been marked in revised Fig 4 to show the distribution of the five final mutants. We also corrected the terms as suggested. In the “primary criteria”, we used a reference range approach establishing percentile values for the entire data set that provides high and low critical values, approximately equating to +/- 2 s.d. but not effect size. The onset time or peak phase which falls in the tails beyond ~+/- 2 s.d. from mean (approximate 5%) were designed outliers, where s.d. represents the standard deviation of differences between the mutants and wild-type control in each center. In the “second criteria”, we have clarified the three criteria we listed in manuscript. The three criteria: 1) 50% of individual mice within a line displayed similar phenotypes as described in previous study (de Angelis et al., 2015; Dickinson et al., 2016; Potter et al., 2016). We applied this criterion to exclude occasional situation. 2) standardized effect size (Cohen's d), where $d=\frac{\overline{x_{m}}-\overline{x_{wt}}}{s}$ is the absolute difference between mutant and baseline means scaled in units of phenotypic standard deviation. A larger [value](https://en.wikipedia.org/wiki/Absolute_value) of effect size always indicates a stronger phenodeviance (de Angelis et al., 2015; Dickinson et al., 2016). Effect size (d) >1.2 suggested that the group difference is large between the mutant mice and the wild-type mice. 3) two-tailed t-test was used to examine the statistical significance (p value), p<0.001 means statistical significance between the mutant line and phenotyping baseline. Mutant mice need to meet all three criteria to be defined as positive.

Q15 Just to make sure that the baby is not thrown out with the bathwater - do the 88 genes that survive primary analysis show enrichment in certain pathways or gene ontologies. Do they show significant enrichment of human GWAS hits for chronotype phenotypes? There has been a slew of these papers.

**Response**: We thank the reviewer for the insightful comments. We retrieved 88 candidate genes at the Sleep Disorder Knowledge Protal (SDKP) website (<http://www.sleepdisordergenetics.org>) (Jones et al., 2019; Wang et al., 2019). We found that *Dbn1*, *Pax4*, *Oxtr*, *Ptpmt1* and *Casc5* showed significant enrichment of human GWAS hits for Morningness phenotypes (p<0.003). However, we cannot further back it up at this stage. So we did not mention this detail in the manuscript.

Q16 Visualization point - The 3D barplots are very hard to interpret. I found myself drawing lines to in 3D space to compare the right bars (https://guides.library.duke.edu/datavis/topten). Showing the data in multiple 2D histograms will be more interpretable. Small multiple plots are generally easier to interpret (https://www.r-bloggers.com/why-you-should-master-the-small-multiple-chart/orhttps://journals.plos.org/ploscompbiol/article?id=10.1371/journal.pcbi.1003833 (Rule 8)).

**Response**: We thank the reviewer for this suggestion. We have modified Fig 4 to multiple 2D histograms.

Q17 Figure 5 – describes the phenotypes of the 5 selected mutants. Please provide a table similar to Figure 3G table for these mutants that clearly describes the effect size and p value (table S11 does not provide both stats).

**Response**: As suggested, we have added screening parameters such as effect size and p value similar to Fig. 3G table in the revised table S11.

**S11 Table. Hits from secondary screen**

|  | **Gene** | **T_on_** | **T_ph_** | ***d_on_*** | ***p_on_*** | ***d_ph_*** | ***p_ph_*** |
| --- | --- | --- | --- | --- | --- | --- | --- |
| **Activity** | ***Slc7a11*** | **10.75±0.89** | **13.00±1.20** | **3.47** | **1.22E-19** | **0.05** | **0.89** |
|  | ***Oxtr*** | **14.29±1.11** | **15.29±1.38** | **0.79** | **3.9E-2** | **0.13** | **0.73** |
|  | ***Rhbdl1*** | **14.38±2.39** | **17.00±2.98** | **2.39** | **1.45E-9** | **0.85** | **2.5E-2** |
|  | ***Spop*** | **13.80±2.62** | **16.00±2.71** | **1.45** | **2.26E-5** | **0.30** | **0.36** |
|  | ***Ctc1*** | **13.30±1.16** | **16.00±1.24** | **5.37** | **1.20E-56** | **1.11** | **5.1E-4** |
| **Food**  **intake** | ***Slc7a11*** | **11.71±1.25** | **14.00±0.93** | **0.73** | **5.6E-2** | **0.41** | **0.25** |
|  | ***Oxtr*** | **11.20±2.28** | **13.8±2.28** | **1.97** | **1.95E-05** | **0.68** | **0.13** |
|  | ***Rhbdl1*** | **13.17±2.79** | **15.33±2.94** | **0.13** | **0.75** | **0.32** | **0.45** |
|  | ***Spop*** | **16.11±2.57** | **17.56±2.70** | **3.89** | **1.05E-23** | **1.15** | **1.5E-3** |
|  | ***Ctc1*** | **14.30±2.31** | **16.60±1.78** | **1.71** | **8.04E-8** | **0.25** | **0.43** |

**T_on_: onset time**

**T_ph_: peak phase**

***d_on_*: effect size for peak phases**

***p_on_*: p value of student’s test of wild type and mutants’ peak phases**

***d_ph_*: effect size for onset times**

***p_ph_*: p value of student’s test of wild type and mutant’s onset times**

Q18. How do these mutants compare in effect size and p value to the validation mutants for the same phenotypes. The Slc7A11 phenotype seems to have a very low effect. It would be helpful to have E and M peaks clearly marked for control and mutant on these plots.

**Response**: Positive control effect sizes varied from 1.2-6.64 for activity onset and 1.24-3.51 for food intake onset. The mutant effect sizes are as follow: 3.47 for *Slc7a11* with lowest p value (1.22E-19); 1.97 for *Oxtr* (p = 1.95E-05); 2.39 for *Rhbdl1* (p = 1.45E-09); 1.45 for *Spop* (p = 2.26E-05); 5.37 for *Ctc1* (p = 1.20E-56). As indicated in previous response, these data have been implemented in revised table S11.

Q19 Figure 6 – The validation of Slc7a11 confirms that this is a very subtle mutant. The onset of activity is slightly disorganized, perhaps advanced. The wheel running DD data has no effect, when mutations with phase advance usually have shorter period. The wheel running actogram that is shown shows that there is slightly more daytime activity in the mutants. This lack of activity consolidation should lead to lowered circadian amplitude. Please check this.

**Response**: As suggested by reviewer #1 and #3, we have now added skeleton photoperiod and Jetlag experiments for further validation. As shown in the revised Figs 6 H-J, and S8 Fig, in response to both a 6 h advance shift under the LD cycle and skeleton photoperiod stimuli under constant darkness, *Slc7a11^tm1a/tm1a^* mice immediately showed phase advance compared with wild type littermates. We also examined the expression profiles of core clock genes and found comparable mRNA levels between *Slc7a11^tm1a/tm1a^* and WT control, suggesting that disruption of *Slc7a11* did not affect the amplitude of these gene expressions. However, we do observe that the phase of those high-amplitude expressed genes *Dbp* and *Per2* are advanced in the SCN of *Slc7a11^tm1a/tm1a^* compared with that of WT. We feel that these results do strengthen our conclusion.

Figure 7 – no comments.

**Reviewer #3:**
This work takes data collected by the IMPC and analyses it for the timing of behavior. This is a wonderful idea, taking advantage of a resource that has many treasures still to be discovered and extending the usefulness of the massive amount of work that has gone into building this program. The validation of the candidate gene, Slc7a11, is also very nicely handled. I do have several comments which I think will make the article more useful to a general readership and boost the scholarship as well.

Q1“However, how internal circadian clocks are entrained to changes in photoperiod remains unclear. “This sentence implies that changes in photoperiod will be addressed. They are not. The authors rather use entrained phase as a phenotypic marker in their screen. Another level of complexity would be to screen on entrainment to different photoperiod.

**Response**: Thanks for bringing this point up. Together with reviewer #1’s comments, we have modified “circadian entrainment” to “circadian misalignment” in the revised manuscript and added circadian entrainment-targeted experiments. We performed jetlag (advance) and skeleton photoperiod experiment to examine the role of *Slc7a11* in circadian entrainment. Modified Fig 6 and S8 Fig have been added to be revised manuscript. In response to a 6 h advance shift of the LD cycle, *Slc7a11^tm1a/tm1a^* mice immediately showed phase advance (S8 Fig). Furthermore, to minimize the masking effect of light, we entrained mice to a skeleton photoperiod with 15-min light arms from clock time 07:45 to 08:00 and clock time 20:00 to 20:15 and complete darkness at all other times. Under these conditions, the activity onset was significantly advanced in *Slc7a11^tm1a/tm1a^* mice compared with wild-type littermates (Fig 6H-J). These data indicate that disruption of *Slc7a11* affects circadian entrainment. Our revised manuscript with additional new results is able to provide more information on how *Slc7a11* mutant mice respond to different photoperiod.

Q2“In addition, activity, feeding, temperature and glucocorticoid signals can also affect the circadian phase of the circadian clock [17-21]. These studies indicate that circadian entrainment is influenced at multiple regulatory levels.”
I think it would be better to say that these are all zeitgebers of the circadian clock and thus by definition, they will impart phase information on their target tissues. Not all of the papers cited actually discuss or systematically probe phase (though they do show phase shifting at least).

**Response**: The reviewer is quite right. We have changed the sentence into ‘In addition, activity, feeding, temperature and glucocorticoid signals can also affect the circadian phase. “These are all zeitgebers of the circadian clock will impart phase information on their target tissues. Circadian misalignment is as a consequence of conflicting signals of these zeitgebers” in p7 line 136-139. For reference, we added a review paper “Physiology of Circadian Entrainment” by DA Golombek, 2010, in which a variety of related studies were comprehensively discussed.

Q3“We hypothesized that mouse mutants with impaired circadian entrainment would result in phase changes in locomotor activity and/or food intake behaviour under the light/dark cycle “
Why? The authors should put this hypothesis into a better context. The story of food entrainment – the one before the molecular era – is inspiring to all scientists! It is an opportunity to help non-chronobiologists understand that chronobiology is not just a time point in the dark and a time point in the light, that it is a robust machinery that regulates our behaviour and physiology systematically. Further, PLoS Genetics is a non-clocks journal and the concept (peripheral oscillators etc.) would benefit from a graphical explanation as well as a few words here.

**Response**:

Thank you for your appreciation of this work. We have now added several sentences to explain the rationale: “Misalignment between different components of the circadian systems and sleep-wake cycles or food intake as a result of genetic, environmental or behavioural factors might be an important contributor to diseases as well as how we can treat and prevent them. The mouse mutants with impaired circadian misalignment would result in phase changes in locomotor activity and/or food intake behaviour under the light/dark cycle. We attempt to provide a systematic genetic approach to understand misalignment between circadian systems and behavioural factors.” in p8 line 163-169.

Q4“The onset time was defined as a transition from rest to steep activity/food intake, while the peak phase was the end of the transition.”
This is not clear. Which point on the transition? For dim light melatonin onset, many groups empirically decide which point of the onset (e.g. 25% level of the upslope of onset) to use.

**Response**: We appreciate the reviewer’s critical comment. To clarify the definition of onset and peak phase, we have modified the text (p 10, line 214-215) and added supplemental figure (S2 Fig) and figure legend in the revised manuscript. Here, we described onset as a transition from rest to active state, like the interim of a piecewise function. We simulate the average raw data from IMPC centers by combining Gaussian functions (for peak phases) and a piecewise function (for onset) (see figure below, S2 Fig) in the process of training set generation. Red dots are average raw data and colorful curve is the fitted curve (S2 Fig). Part of data before E peak phase (data between two red dashed lines in S2 Fig) is fitted by a piecewise function (blue curve in S2 Fig A). Dividing point of two stages is indicated by two black arrows (S2 Fig A and B). The first data entering transition is identified as onset (S2 Fig A, green arrow) and peak phase is indicated by blue arrow (S2 Fig A).

Q5 The results for the onset time and peak phase of activity and food intake for each centre were determined by SyncScreener (Figs 2C and 2D, S3 and S4 Tabl “
I think it would be worth graphing the peak and onset values for activity and food intake for the 5 centers. The data are shown here and we get an impression of the differences but we should see this graphed rather than these figures and the table in the suppl materials. Graphical representation would be helpful to appreciate the variance.

**Response**: The reviewer’s point is well taken. The figure below presenting onset and peak values for activity and food intake from the five centers were implemented as revised Fig 2E-H to clarify this point.

Q6 “We reasoned that the onset time was easily recognized by visual assessment due to an obvious steep ascension from an inactive state, whereas peak phase sometimes displayed as a plateau, which may lead to variability in peak phase identification.”
Therefore the onsets are used? It is not clear what the conclusion is here and if a decision was taken. If the peak is unstable (I have seen this also and also prefer onsets) a measure called Center of Gravity can also be used.

**Response**: We appreciate your comments. Here, we want to describe a comparison between onset and peak phases. We acknowledge that peak phase is less stable than onset, and we did not find hits from peak phase in this screening. However, we kept this parameter for screening in order to discover possible mutants which greatly affect the properties of peak phase like to *Zbtb20* KO mice. To avoid confusion, we have deleted that sentence in the revised manuscript.

Q7“Finally, to validate our predictive models and parameters, we used several known circadian mutant lines to evaluate the circadian parameters”
Two things here: Before going into this, we would like to know about the variance (beyond the nice heat maps) in the controls. How does this look in comparison to wheel running data in the same strain? I think it would be important to not just show the pooled data from the sites but to show the variation of all individuals. What is the onset with SD? Before looking at the mutants, we would like to know this.

**Response**: Thank the reviewer’s comments. Our new result in revised Fig 2E-H showed the variance of onset and peak phase in each center. As explained above, peak phase is less stable than onset. Although we did not find hits for peak phase from 750 mutant lines, we kept this parameter for screening in order to discover possible mutants which significantly affect the properties of peak phase.

We didn’t perform a comprehensive study to compare wheel running test and IC data since we could only obtain IC data from IMPC. From our experience and other colleagues’ studies, voluntary wheel-running activity also alters circadian rhythms in mice. For example, wheel-running activity shortens the period of the activity (Harrington et al., 2007) and accelerates re-entrainment to shifted light-dark cycles (Leise et al., 2013). Wheel-running activity delays specifically the phase of the liver (Pendergast et al., 2014). In contrast, the activity measured by metabolic cage are based on infrared beam breaks with X, Y, Z plane and are not interfered by exercising and rewarding-related behavior, thus this method can better reflect the phase phenotype than wheel-running. However, mice in the metabolic cages without bedding and environmental enrichment have been associated with induction of stress or discomfort so that it could not be used for long term recording.

Q8 How does this data on clock mutants compare to what is published?
“delayed onset and peak phase of both food intake and activity compared to wild-type mice at TCP (Figs 5C-5F), suggesting that circadian robustness might be impaired.“
I do not agree with this statement/conclusion. Can the authors back it up?
**Response:** We totally agree and have deleted that sentence.

Q9 Rhbdl1+/tm1a mutant mice also displayed vision defects“
Very nice observation showing the power of this screen to reveal entrainment mutants. (In Neurospora there have been mutants reported with no difference in period but rather in entrainment.)
**Response**: Thank you for your appreciation of this screen.

Q10 The Oxtrtm1a/tm1a mice exhibited a trend towards more daytime activity and food intake tendency” Is this backed up somewhere with data?

**Response**: Thanks for bringing this point up. As suggested, we quantified the day-night activity and food intake ratios in the revised S5 Fig. The figure below represents the daytime activity and food intake of *Oxtr^tm1a/tm1a^* mice compared with wild-type littermates, suggesting that the *Oxtr^tm1a/tm1a^* mice exhibit a trend towards more daytime activity but not the food intake.
